# Recent advances in lanthanide-doped up-conversion probes for theranostics

**DOI:** 10.3389/fchem.2023.1036715

**Published:** 2023-02-09

**Authors:** Danyang Xu, Chenxu Li, Wenjing Li, Bi Lin, Ruichan Lv

**Affiliations:** Engineering Research Center of Molecular and Neuro Imaging, Ministry of Education, School of Life Science and Technology, Xidian University, Xi’an, Shaanxi, China

**Keywords:** lanthanide-doped, probes for theranostics, up-conversion, up-conversion luminescence, biomedicine

## Abstract

Up-conversion (or anti-Stokes) luminescence refers to the phenomenon whereby materials emit high energy, short-wavelength light upon excitation at longer wavelengths. Lanthanide-doped up-conversion nanoparticles (Ln-UCNPs) are widely used in biomedicine due to their excellent physical and chemical properties such as high penetration depth, low damage threshold and light conversion ability. Here, the latest developments in the synthesis and application of Ln-UCNPs are reviewed. First, methods used to synthesize Ln-UCNPs are introduced, and four strategies for enhancing up-conversion luminescence are analyzed, followed by an overview of the applications in phototherapy, bioimaging and biosensing. Finally, the challenges and future prospects of Ln-UCNPs are summarized.

## 1 Introduction

Rare earth-based nanomaterials have attracted attention due to their superior fluorescence properties. Because of their dense energy levels that enable complex electronic transitions, rare Earth elements have become a treasure house of luminescence (Li et al., 2015a; Binnemans, 2015). Rare earth-based nanomaterials require specific wavelength of excitation to photoluminesce (Tsang et al., 2015; Adachi, 2018; Quan et al., 2018; Mintz et al., 2019; Mir et al., 2020; Gierschner et al., 2021). The emission can be characterized as up-conversion or downconversion luminescence (Reddy et al., 2018). For example, Ln- UCNPs can be excited by infrared light to emit visible light. If modified properly, they can become good candidates for tumor diagnosis and treatment.

Phototherapy (PT) (Li et al., 2018; Lan et al., 2019; Zhen et al., 2019; Xie et al., 2020), as a new type of treatment with good targeting and low toxicity, is often classified as photodynamic therapy (PDT) or photothermal therapy (PTT). Phototherapy is gradually becoming an important supplement for tumor treatment. Therefore, Ln-UCNPs can serve as a substrate for composition with other materials for phototherapy, to aid surgical navigation, to enhance the precision of imaging, and for efficient treatment (Shanmugam et al., 2014; Liu et al., 2019a).

In addition to therapy, Ln-UCNPs also have potential for use in bioimaging and biosensing (Zhang et al., 2016; Zhang et al., 2019). Functionalized Ln-UCNPs can be applied in Computed Tomography (CT) and Magnetic Resonance Imaging (MRI) among others, which adds value to the bioimaging field (Zhang et al., 2011; Sun et al., 2013; Li et al., 2019). Regardless, Ln-UCNP-based biological imaging has shortcomings and there is significant room for improvement. Ln-UCNPs also perform well in biosensing (Guo et al., 2016; Mahata et al., 2017). Ln-UCNPs can be used as pH and temperature sensors as well as gas and DNA sensors, to name a few, due to temperature sensitivity and other optical properties. It is foreseeable that the use of Ln-UCNPs will expand in the sensor field (Wang et al., 2014a; Cui et al., 2014; Dong et al., 2015; Pan et al., 2017).

This manuscript reviews research on the synthesis, luminescence modulation, and the latest achievements in phototherapy, bioimaging and biosensing of Ln-UCNPs. Synthetic methods are summarized in Section 2, research on luminescence modulation is outlined in Section 3, and then applications for Ln-UCNPs in phototherapy, bioimaging and biosensing are introduced in Section 4. Finally, the current challenges of Ln-UCNPs and their prospects for the future are discussed.

## 2 Synthesis methods of Ln-UCNPs

Many challenges concerning the morphology and performance of Ln-UCNPs must be addressed for successful application in biomedicine. Fortunately, many kinds of Ln-UCNPs have been synthesized that enable the potential for applications. Rare Earth luminescent materials have attracted much attention because of their flexibility and easy modulation. Different nanoparticles can be synthesized by controlling the reaction conditions; these include the temperature, pH, precursor concentrations, etc. Researchers have successively realized several luminescence mechanisms such as excited state absorption (ESA), energy transfer up-conversion (ETU), cooperative sensitization up-conversion (CSU), photon avalanche (PA), and energy transfer-mediated up-conversion (EMU). This section introduces the most common methods, specifically thermal decomposition and hydrothermal/solvothermal, as well as several other synthetic approaches. A brief comparison of these methods is provided in Table 1.

**TABLE 1:** Typical methods of Ln-UCNPs synthesis (Li and Lin, 2010; Du et al., 2011; Lin et al., 2012).

Method	Morphology	Reaction condition	Product stability	Synthetic cost
Thermal decomposition	Controllable	Harsh	Stable	Higher
Hydrothermal/solvothermal	Controllable	Simple	Stable	Lower
Co-precipitation	Uncontrollable	Simple	Stable	Lower

### 2.1 Thermal decomposition method

The thermal decomposition method is the most facile to create high-quality Ln-UCNPs. Thermal decomposition is the process of pyrolyzing organometallic precursors in an organic solvent under an oxygen-free environment. In general, the precursors are organic salts of rare Earth ions, such as trifluoroacetate and acetate among others. Octadecene (ODE) is the most frequently used high-boiling organic solvent. Oleic acid (OA) and oleylamine (OM) can serve dual roles as solvents and as ligands that adsorbed onto the nanoparticles to control their size and shape. It is worth noting that the crystal nucleation and growth process that control the nanoparticle uniformity can be adjusted with the temperature, heating rate, and precursor concentrations during synthesis.

Yan and co-authors first synthesized LaF_3_ crystals by thermal decomposition using a single-source precursor (Zhang et al., 2005). Later they pioneered the use of multi-source precursors to prepare high quality nanocrystals through thermal decomposition, which greatly promoted the development of Ln-UCNPs (Yin et al., 2010). Murray and collaborators studied the influence of reaction time and the ratio of sodium to lanthanide precursors on the morphology of β-NaYF_4_-based Ln-UCNPs in detail, thereby obtaining beautiful rare earth-based nanocrystals (Ye et al., 2010). We applied LnCl_3_ (Ln = Y and Er) as precursors in an OA and ODE mixture that was heated to a high temperature, next NH4F and NaOH were added to obtain core nanoparticles of NaYF_4_:Er. Finally Lu(CF_3_COO)_3_ and CF_3_COONa mixed with NaYF_4_: Er were further processed under high temperature and oxygen-free environment to obtain stable NaYF_4_:x%Er@NaLuF4 (Feng et al., 2018).

Ln-UCNPs synthesized using the thermal decomposition method are highly monodisperse and uniform in shape. They also display greater up-conversion emission intensity; however, the syntheses are complicated and the products are lipophilic and not stable, which limits the application of thermal decomposition in Ln-UCNPs synthesis.

### 2.2 Hydrothermal/solvothermal method

The hydrothermal/solvothermal method is facile for synthesizing Ln-UCNPs under mild conditions at a low cost. Generally, hydrothermal syntheses are carried out in a reactor to provide a high-pressure environment. Simple rare Earth nitrates or chlorides are used as precursors for the preparation of Ln-UCNPs. The most common solvents are water, ethanol, glycol and other simple hydrophilic inorganic/organic solvents (Rafique et al., 2019). Li and co-workers introduced oleic acid into solvothermal synthesis. As an oleophilic organic solvent, it broadened the range of materials and structures that can be realized using this method (Wang et al., 2005). The selection of organic additives has a significant impact on the morphology and size of the product. Hydrophilic organic ligands that can adhere to the surface of products and inhibit particle aggregation can endow the nanocrystals with good hydrophilicity, biocompatibility, and functionalizability with biomolecules. It was reported that ethylene diamine tetra acetic acid (EDTA), polyvinyl pyrrolidone (PVP), hexadecyl trimethyl ammonium bromide (CTAB) and polyacrylic acid (PAA)/poly ethylenimine (PEI) as additives improve the morphology and dispersion of Ln-UCNPs (Mousavand et al., 2010; Li et al., 2020a). A mixed solution of Ln(NO_3_)_3_, Y:Yb:Tm = 70:30:0.5, was used as a rare Earth precursor with EDTA as an organic additive. NaYF_4_: Yb^3+^, Tm^3+^ were synthesized by high temperature and pressure in a reactor (Xu et al., 2020). The advantages of hydrothermal/solvothermal method include the potential for large-scale production. Moreover, the reaction is always carried out under closed conditions without harmful gas leakage, so it is environmentally friendly and non-toxic. However, these conditions also make it impossible to observe the crystal growth process, which impede the studies into the reaction mechanisms.

### 2.3 Co-precipitation methods

**FIGURE 1:**
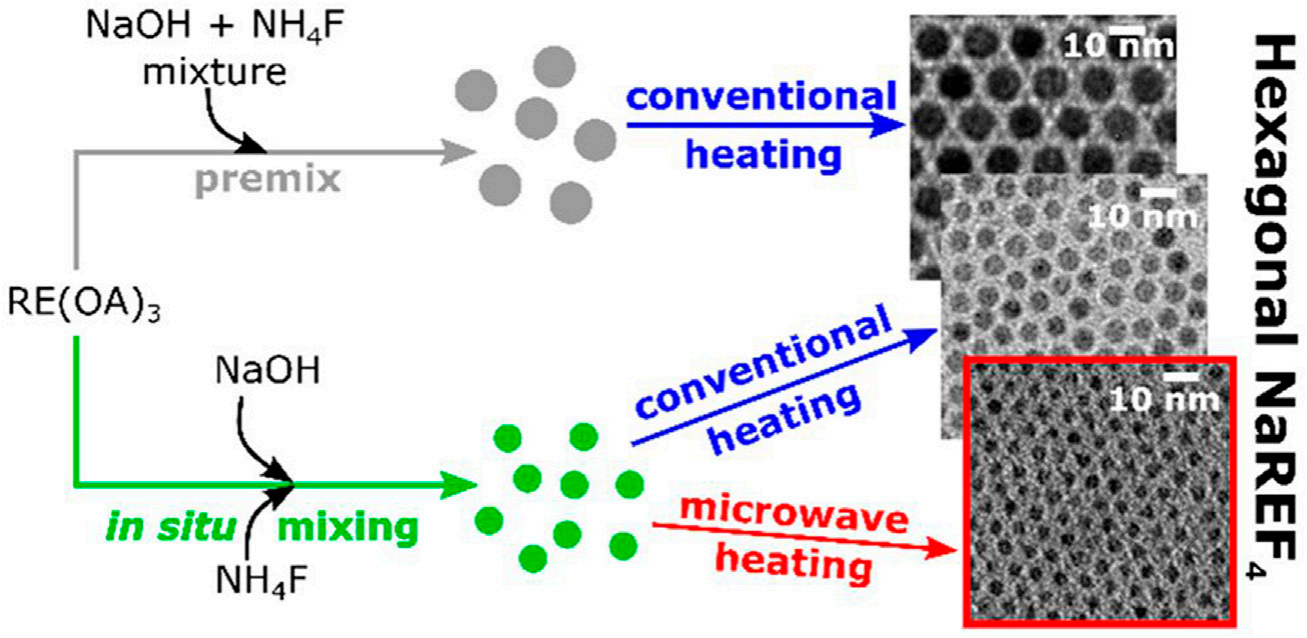
Mixing and high-temperature heating steps are important processes in the controlled thermal coprecipitation synthesis of sub-5-nm Na(Gd−Yb)F4:Tm. [Reprinted with permission from Ref. (Amouroux et al., 2019) Copyright 2019: American Chemical Society].

The products of the co-precipitation method are usually chemically homogeneous and uniformly distributed; however, the surfaces are rough and difficult to control. Coudret et al. obtained ultra-small Na(Gd-Yb)F_4_:Tm through an improved co-precipitation method (Figure 1). In addition to the synthesis of specific precursors and microwave-assisted heating, the influence of oleic acid content on heating efficiency was discussed (Amouroux et al., 2019). In 2014, Liu and co-workers demonstrated a simpler co-precipitation method to prepare core-shell NaGdF4 nanoparticles doped with luminescent lanthanide ions that did not require precise control of the feeding rate of shell precursors. However, the large core-shell nanocrystals had a low up-conversion efficiency (Wang et al., 2014b). Jin and co-workers synthesized a near-infrared (NIR) fluorescence “turn-on” kit based on rare Earth ion-doped nanoparticles and gold nanoparticles, which created an HIV-1 based DNA detection system that was simple, homogeneous, and highly selective (Zhou et al., 2019). Chen and co-workers were the first to report the synthesis of CaS:Eu^2+^,Sm^3+^ near infrared photostimulated luminescence nanocrystals by a high temperature co-precipitation method. The materials exhibited a fast response to stimuli over 800–1,600 nm (Gao et al., 2019). In conclusion, the application of the co-precipitation method creates products that have biomedical applications; however, they are limited by defects. Regardless, this simple process is conducive to industrial production.

### 2.4 Other methods

In addition to the processes discussed previously, several novel methods have been developed for the synthesis of Ln-UCNPs. For instance, microemulsions and ionic liquids have been applied, and Ln-UCNPs have been prepared using microwave heating. The microemulsion method creates materials in droplets that contains the surfactant, cosurfactant, solvent and water. Few product nanoparticles are prepared this way; furthermore, the dispersion is poor because the growth of the particles is affected by the size of the micelle. Li and colleagues reported a method of microemulsion assisted synthesis of functionalized Ln-UCNPs. The nanoparticles was synthesized in a heterogeneous oil-water microemulsion phase, and the product had a high cross-section and adjustable pore size (Dai et al., 2020).

Microwave synthesis has unique properties, including increased speed and heating efficiency, which contributes to energy savings and environmental protection. It was reported that NaGdF_4_:Yb, Er can be directly synthesized using a microwave digestion/extraction system; furthermore, the produced have good biomedical application prospects (Li et al., 2012).

## 3 Up-conversion luminescence modulation

Key issues concerning the optical properties of Ln-UCNPs include the small absorption cross-section, fluorescence quenching, and the electronic structure that may result in multiple emission lines despite excitation at a single wavelength. As a result, Ln-UCNPs should be classified according to the requirements of applications (Li et al., 2015b). In recent years, a series of Ln-UCNPs with enhanced up-conversion luminescence (UCL) have been obtained by adjusting the composition and structure. This provides a deeper understanding of the characteristics and nature of rare Earth luminescence and broadens the potential for applications (Wu et al., 2016; Garfield et al., 2018).

### 3.1 Species and concentration of doping ions

Up-conversion is the process of converting low-energy photons into high-energy luminescence, which relies on energy transfer within the 4f electron manifold of the luminescent ions. Luminescence mechanisms include excited state absorption (ESA), energy transfer up-conversion (ETU), and cooperative sensitization up-conversion (CSU). The ESA process occurs within a single ion that continuously absorbs photons to create upconverted luminescence. The ETU process involved in a pair of neighboring rare Earth ions, a sensitizer as energy donor, and an activator as energy acceptor. After excitation, the ground state sensitizer ion becomes electronically excited, the energy from which is transmitted to activators. In turn the activator ion transitions to a higher electronic state, and then luminesces to the ground state. The CSU process requires at least three ion centers. Two of them are the same element that act as sensitizers. They can interact with activators simultaneously in the excited state, which in turn transfers energy to activators. Consequently, the doping of rare Earth ions is closely related to the up-conversion luminescence ability of the nanoparticles, and the luminescence intensity can be regulated by adjusting the species and concentration of rare Earth ions.

Ln-UCNPs are composed of activators and sensitizers within a matrix. For up-conversion, the matrix must have excellent optical properties and stability. The most studied are fluoride materials NaYF_4_, LaF_3_, LiYF_4_, and NaGdF_4_ among others (Wang et al., 2011; Liu et al., 2012; Wang et al., 2014b; Zhang et al., 2015). To achieve up-conversion luminescence, the energy difference between at least three adjacent energy levels of the activator should be in close proximity. Therefore, Er^3+^, Tm^3+^ and Ho^3+^ are the most important due to their step-like energy levels. Sensitizer ions are usually introduced due to the fact that the luminous efficiency of single-doped Ln-UCNPs is not high. Yb^3+^ has become the most common sensitizer due to its unique energy level structure (Han et al., 2014).

We synthesized highly doped Er^3+^ core-shell nanoparticles of NaYF_4_:x%Er@NaXF_4_ (x = 5, 25, 50, and 100; X = Lu and Y). The effect on luminescence as a function of Er^3+^ concentration was studied. There was a significant enhancement of red emission at 100% Er^3+^ doping (Feng et al., 2018). In a sensitizer and activator co-doped system, the concentration of activator generally does not exceed 5% while the sensitizer concentration is relatively high, on the order of 15%–40%. The emission of Yb^3+^/Er^3+^ co-doped systems range from 510 nm to 560 nm (green) and 640 nm–670 nm (red). This system can emit with multiple colors as a combination of green and red can produce yellow light. Er^3+^/Yb^3+^ co-doped Na_0.5_Gd_0.5_Mo_O4_ was reported to emit with characteristic peaks at 531/552/667 nm; furthermore, the intensity of UCL increased with higher Yb^3+^ concentration over a certain range (Du et al., 2016). Similarly, the intense emission of the Yb^3+^/Tm^3+^ co-doped system was in the blue region, while the Yb^3+^/Ho^3+^ co-doped system mostly emitted green light with improved efficiency due to the sensitizer. Rare Earth ions have further utility. For example, multi-doped systems empower enhanced luminescence modulation. Liu et al. reported white light emission from Er^3+^/Tm^3+^/Yb^3+^ triple-doped SrLu2O4. The Yb^3+^ concentration and pump power can be tuned to modulate the emission color. The optimal doping ratio of 0.5% Er^3+^, 0.5% Tm^3+^, and 20% Yb^3+^ created the best balance of red, green and blue luminescence to produce bright white light emission (Liu et al., 2018a). Dammak and coworkers designed a white light modulation system, Yb^3+^/Er^3+^/Tm^3+^ doped GdPO_4_ at a high concentration of Yb^3+^ (35%mol), which emitted white light with excitation by an appropriate laser power (Hassairi et al., 2018). β-NaYF_4_: Yb^3+^, Ho^3+^, Tm^3+^ triple-doped Ln-UCNPs can create multicolor UCL by adjusting the doping concentration and excitation power, which resulted in both white and red light output (Gao et al., 2017).

### 3.2 Surface plasmon resonance modulation

**FIGURE 2:**
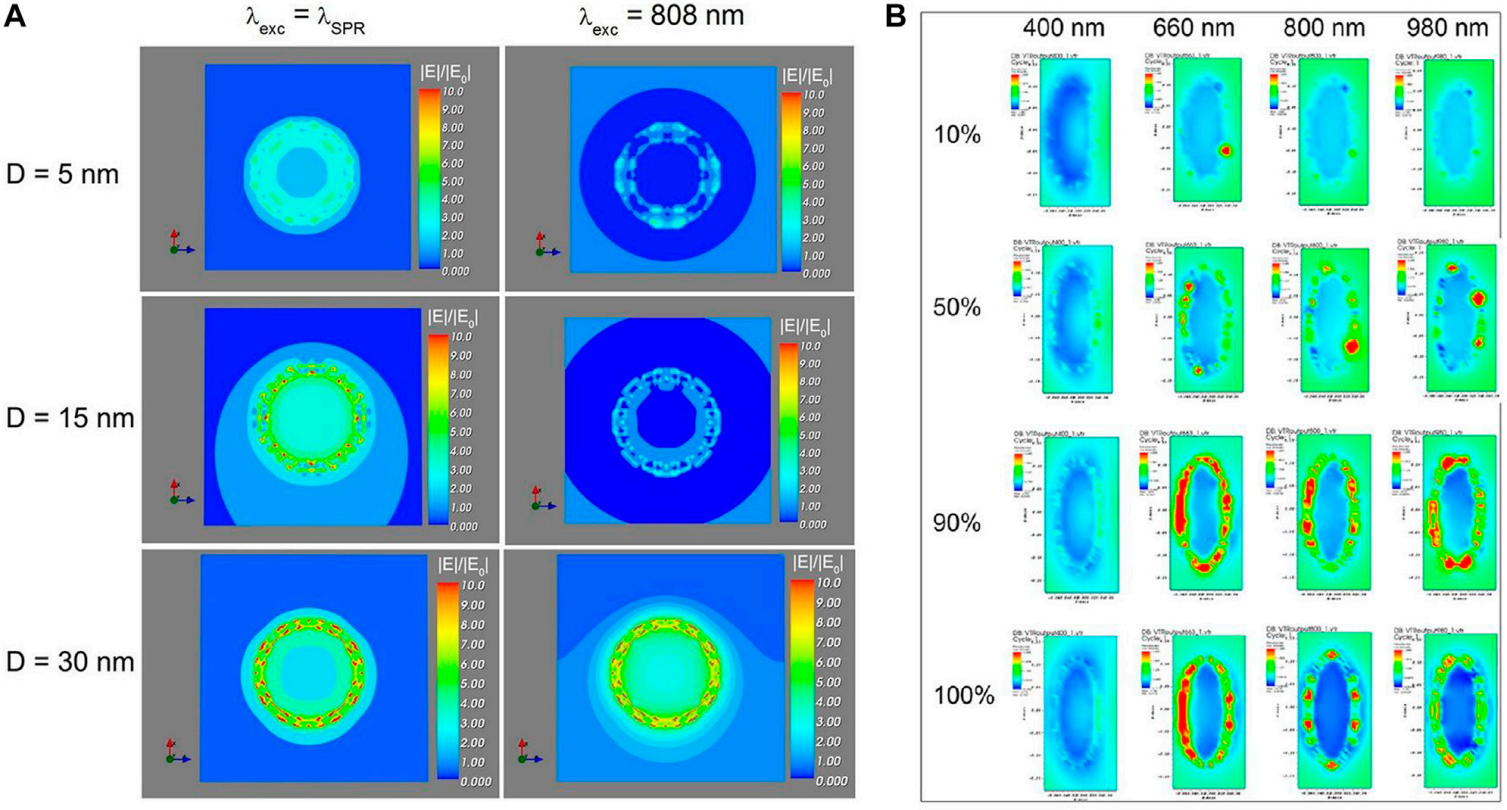
**(A)** Simulation of the electric field strength (|E|/|E0|) of Ln-UCNPs@mSiO2-Au NPs (15 nm–D–5 nm geometry) under irradiation at λexc = λSPR and λexc = 800 nm, with different silica spacers with thickness D of 5, 15, and 30 nm |E|/|E0| is the enhancement factor. The electric field is amplified as |E| > |E0|. [Reprinted with permission from Ref. (Lv et al., 2018a) Copyright 2018: Lv et al. (2018a)]. **(B)** DDSCAT simulation results. Extinction spectra of the corresponding electronic strength images of SPS@Au. [Reprinted with permission from Ref. (Lin et al., 2020) Copyright 2020: American Chemical Society].

Metal nanoparticles exhibit a strong plasmon resonance intensity due to electron delocalization over a large surface area. The surface plasmon resonance (SPR) effect will occur when the frequency of the incident photon is the same as the surface plasma oscillation. At this time, a strong local electric field is generated around the metal nanostructure, which further increases the excitation and radiation decay rates of Ln-UCNPs and thus enhances the up-conversion luminescence. UCL can be enhanced by SPR (Thariani and Yager, 2010), which prompted us to theoretically explore the effects of metal nanoparticles on Ln-UCNPs luminescence using the Discrete Dipole Approximation (DDA). The DDA algorithm can calculate the electromagnetic properties of nanoparticles as a function of shape. We synthesized La2O3:Yb/Er@Au, the gold coating of which significantly enhanced the emission intensity of La_2_O_3_:Yb/Er@Au by 16.8 times. Experiments demonstrated that the amount of Au added affects the particle spacing and the intensity of UCL. The increase in the electric field simulated by DDA was in good agreement with the observations made in the presence of an Au coating (Lv et al., 2014a). Light exposure and heating of the metal were shown to result in drug release in Na_5_Lu_9_F_32_:Yb/Er@Au. Both DDA simulation and experimental studies of Ln-UCNPs revealed that the coating of Au enhanced the absorptivity, which in turn resulted in a temperature increase upon excitation that facilitated the release of doxorubicin (DOX). Therefore, this system can be used for controlled drug release by the photothermal effect (Lv et al., 2014b). In the first two systems, we only performed electromagnetic calculations on single Au nanoparticles. DDA was used for the first time to perform extinction calculations on the rare earth-metal model of Ln-UCNPs@SiO_2_-Au (Figure 2A) (Lv et al., 2018a). The results revealed the potential to use DDA to guide the construction of rare earth-metal composite materials.

Although considerable progress has been made, many issues remain to be addressed. In the simulations discussed above, gold shells were used to simulate gold coatings. To obtain more accurate results, the Au shells were replaced with randomly distributed gold spheres to simulate Ln-UCNPs@mSiO_2_-Au. Both DDA simulation and experiments demonstrated that the presence of 5 nm Au spheres results in enhancements; however the experimental results on 20 nm Au sphere were inconsistent with the DDA simulations due to insufficient uniformity and aggregation. Regardless, the overall accuracy of the model on the modulation of the luminescence was improved (Lv et al., 2020). Next, we synthesized Au/Ag@Ln-UCNPs, and used DDA to simulate the enhancement on UCL due to the presence of gold nanocages of different sizes and thicknesses. Optical characterizations revealed that the luminous intensity of Au/Ag@Ln-UCNPs is twice that of Ln-UCNPs alone. In addition, Au/Ag@Ln-UCNPs were also effective for PTT/PDT treatment of tumors (Liu et al., 2019b). Additionally, Ln-UCNPs@SiO_2_@Au (SPS@Au) was synthesized and coated with ZnPc (zinc phthalocyanine) to produce SPS@Au/ZnPc. DDA was used to simulate the extinction of SPS@Au/ZnPc as a function of gold particle content (Figure 2B), the results from which revealed enhanced luminescence properties (Lin et al., 2020).

The finite difference time domain (FDTD) method can be used to simulate the enhancement of UCL due to metal SPR. Song and coworkers used FDTD to simulate NaYF_4_: Yb^3+^, Er^3+^ up-conversion luminescence in the presence of a gold rod. Single-layer gold nanorod (GNRs) and Ln-UCNPs were assembled on both sides of an isolating MoO_3_ layer to demonstrate a luminescence enhancement effect of more than 35 times (Yin et al., 2016). Zhou and coworkers used FDTD to model Ag@SiO_2_ to calculate the optimal Ag particle size and silicon layer thickness. The prepared Ag@SiO_2_@YF_3_:Ho^3+^ nanoparticles exhibited better luminous intensity (Xu et al., 2019). Although enhancements are realized using the current strategy of metal modulation to enhance UCL, the effects are actually quite modest. Methods to achieve greater optical enhancements is a problem to be solved in the future.

### 3.3 Algorithm optimization

A large number of experiments are usually required to determine the optimal doping concentration for Ln-UCNPs.

However, computer technology has revolutionized the traditional screening method. Using algorithms to guide this process can greatly simplify experimental realization. The doping optimization of rare Earth luminescent powder is essentially a combination optimization problem. A product can be designed on a computer and then experimentally realized; these steps are repeated to obtain a material with the best properties. In this process, heuristic algorithms based on natural body algorithms, have been used to guide the synthesis of rare earth-doped luminescent powders with high quantum yield. Heuristic algorithms include the genetic algorithm (GA), particle swarm optimization (PSO), and simulated annealing (SA) (Hopper and Turton, 2001; Hamza et al., 2017; Ozdemir and Karaboga, 2019). GA is a global random search optimization algorithm that uses the “survival of the fittest” mechanism to derive relationships between the material properties and the mole percentage of each raw material, and is often used to guide chemical synthesis optimization problems (Katoch et al., 2021). PSO simulates “bird predation.” The algorithm is easy to understand and program, has global search ability; as a result it has been applied in the field of combinatorial chemistry. Annealing is the process in which the temperature of the system decreases with time (Thangaraj et al., 2011). SA continuously iterates a simulated annealing process to find a solution that meets design specifications (Suman and Kumar, 2006). This algorithm has not yet been applied to combinatorial chemistry problems. Therefore, the most widely used optimization algorithm is the genetic algorithm (Lv et al., 2018b).

**FIGURE 3:**
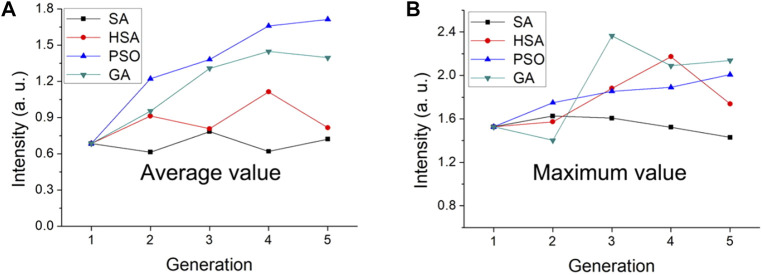
Comparison of SA, HSA, PSO, and GA algorithms to the final average and maximum luminescence intensity. Variance of **(A)** all concentrations and **(B)** the Ce/Tb concentrations in different generations. [Reprinted with permission from Ref. (Lv et al., 2019) Copyright 2019: American Chemical Society].

Our group has studied the pros and cons of SA, the improved SA of harmony search, PSO, and GA for guiding the synthesis of luminescent powders (Figure 3). The efficacy of the four algorithms were evaluated by taking the same first-generation luminescent powder as the starting point for optimization and performing 5 iterations. SA had no obvious optimization effect; however, better results were obtained when combined with the harmony search. The genetic algorithm was better than PSO; however, generational analysis found that the brightness of the luminous powders as guided by PSO was more gradual (Lv et al., 2019).

In recent years, it was reported that the regression equation was built through the experimental results, which showed the relationship between luminescence and doping concentration. Then genetic algorithm was used to get the doping concentration corresponding to the maximum luminescence intensity (Sun et al., 2015). Coincidentally, Zhao et al. (2019) used the genetic algorithm to modulate Er^3+^/Yb^3+^ co-doped Ba5Gd8Zn4O21, resulting in efficient red light emission.

### 3.4 Dye sensitization

Ln-UCNPs are challenged with weak absorptions and narrow optical excitation regions. These issues can be addressed using the dye sensitization strategy in which organic near-infrared chromophores are coordinated to the surface of Ln-UCNPs. Examples include IR783, IR806, IR808, and indocyanine green (ICG) which act as “antennas” to capture photons and transfer energy to the up-conversion rare Earth particles to improve UCL (Hazra et al., 2018). In 2012, Zou et al. (2012) used IR806 to sensitize β-NaYF_4_: Yb, Er for the first time. In this study, the intensity of up-conversion luminescence *via* excitation with an 800 nm laser increased by 1,100 times compared to that realized from excitation at 980 nm. This work prompted widespread application of this strategy for up-conversion luminescence enhancement. Han and coworkers synthesized (β-NaYF_4_: 20% Yb^3+^, 2% Er^3+^) Ln-UCNPs without hydrophobic organic ligands, and then explored the best ratio of near-infrared dyes to Ln-UCNPs to enhance luminescence. They coordinated IR783, IR808, IR820 and IR845 to Ln-UCNPs, and found enhancements of 80, 200, 70 and 10, respectively. To explore the sensitization effect of multiple dyes, IR783 and IR845 were simultaneously coordinated to the same Ln-UCNPs. The multidye-sensitized Ln-UCNPs showed a wider wavelength range and were applied for orthogonal bioimaging (Wu et al., 2015). Chen et al. proposed a new concept of energy-cascaded up-conversion (ECU) through the design of an IR808-sensitized core/shell Ln-UCNPs. IR808 was coordinated to the Ln-UCNPs and acted as an antenna to collect excitation energy that was transferred to Nd^3+^, Tm^3+^ and Yb^3+^ through a multi-step process. This approach achieved a high up-conversion quantum efficiency of 19% (Chen et al., 2015).

Although the sensitization strategy can significantly enhance UCL, the dyes are quenched in water which greatly limits potential applications. Researchers have explored the synthesis of more stable and brighter dye-sensitized Ln-UCNPs. It was reported that Ln-UCNPs sensitized with Cy7 enhanced UCL in ethanol by ∼30 times, although the effect was limited in water by ∼2 times. To address this problem phosphatidylcholine was introduced to improve water dispersibility, which resulted in an increase of 17 times efficiency and enabled lymphatic imaging (Zou et al., 2016). Liu and coworkers revealed that quenching of dye sensitized Ln-UCNPs in water is the result of dye aggregation. Generally, near-infrared dyes are coupled to Ln-UCNPs through chemical coordination; in contrast, Liu and coworkers constructed dye-sensitized systems through hydrophobic interactions. The amphiphilic molecule DSPE-PEG (1,2-distearoyl-n-glycerol-3-phosphoethanolamine-N- [(polyethyleneglycol)-methoxy]) was used to coat the dye Car-Cl. As a result, the sensitization was improved 85 times in water. Unfortunately, aggregation-induced quenching was nonetheless observed at high concentrations.

**FIGURE 4:**
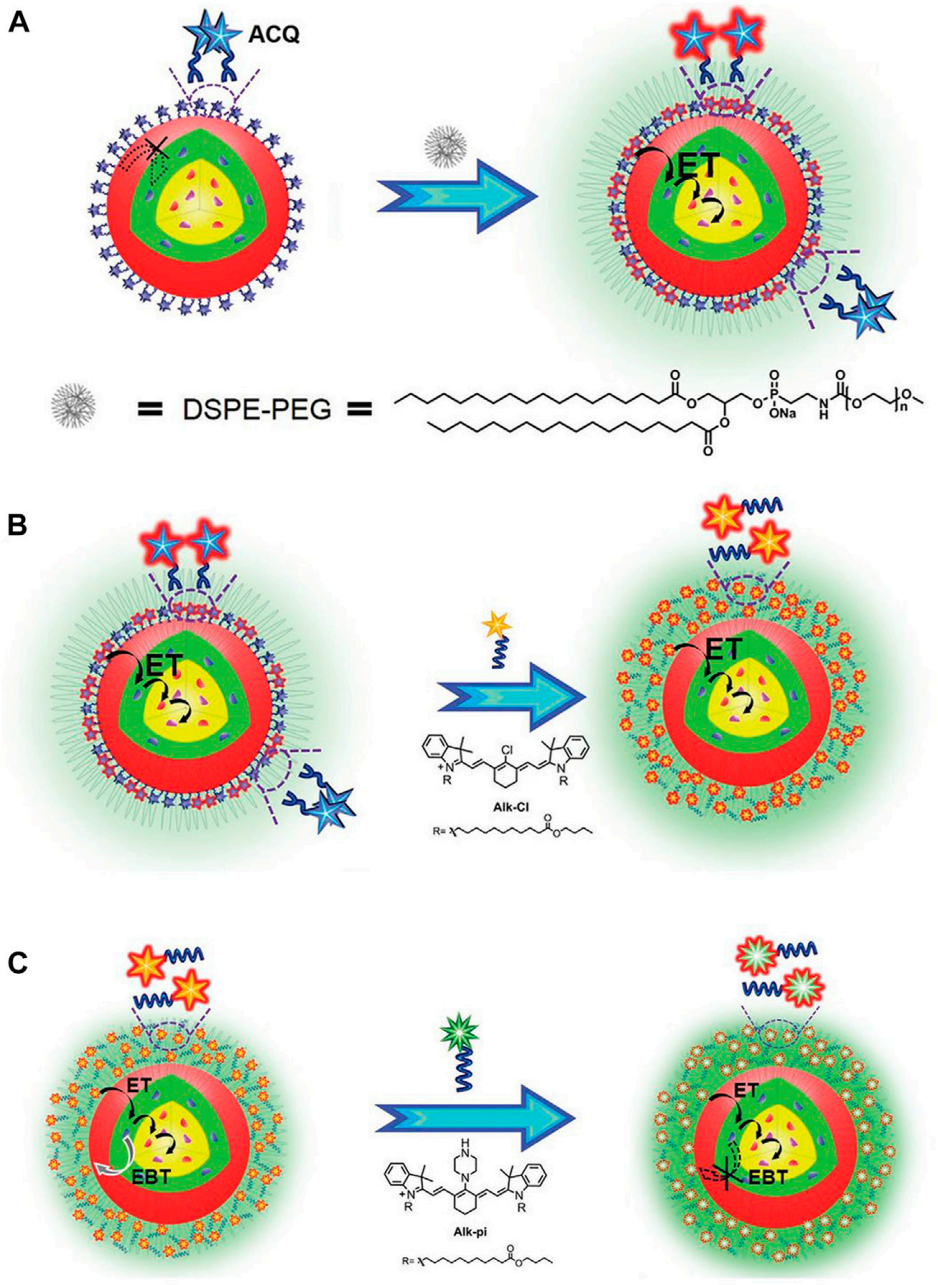
**(A)** Schematic illustration of the alleviation of Aggregation-Caused Quenching (ACQ) and promotion of dye sensitization in aqueous phase by coating with DSPE-PEG. **(B)** Schematic illustration of improving dye-sensitization performance through eliminating ACQ of dye molecules. **(C)** Schematic illustration of improving dye sensitization performance through alleviating EBT from Nd^3+^ to dyes. [Reprinted with permission from Ref. (Liang et al., 2020a) Copyright 2020: John Wiley & Sons, Inc.].

To further eliminate strong coordination between the dye and rare Earth ions, Liu and coworkers used two long hydrophobic alkyl chains to replace the carboxyl group of the dye Car-Cl to produce a new dye (Alk-Cl), the use of which achieved enhancement of UCL of 215 times (Figure 4) (Liang et al., 2020a). In addition, NIR-II dye (IR-1061) was used to sensitize core-shell Ln-UCNPs and solve the problem of quenching in water. It was worth noting that the purpose of this research was not to enhance up-conversion, rather, to obtain a strong near-infrared signal. Application of this strategy resulted in an increase in the 800 nm emission of Tm^3+^ ions by 2.83 times. Furthermore, the near-infrared emission had a deeper tissue penetration depth, which is more conducive for biological imaging compared to visible emitters (Hazra et al., 2018). Although Ln-UCNPs have multicolor emission, it should be noted that red light has the advantages of deep penetration depth and imparts minimal damage to tissues in biological application.

### 3.5 Surface modification

During the synthesis of nanoparticles, long-chain polymers like oleic acid always were wrapped on the surface of Ln-UCNPs. In this way, the stability and luminescence intensity in aqueous are decreased compared with organic solvents, which limits the application in organisms. The following methods are usually used to deal with synthesized Ln-UCNPs. 1) Acid or excess ethanol was added. The ligands on the surface of Ln-UCNPs can be removed under the ultrasound. Capobianco and co-workers successfully removed the oleic acid ligands on the surface of NaGdF_4_: Yb^3+^,Er^3+^ by the means of acid treatment (Bogdan et al., 2011) 2). The hydrophobic ligands were replaced by hydrophilic ligands, such as PEI, PAMAM, and PAA, to increase the water solubility of the nanoparticles. (Kovalenko et al., 2009; Kovalenko et al., 2010; Tangirala et al., 2010) 3) Inorganic material shells were coated on the surface of Ln-UCNPs, such as SiO_2._ This method can improve the water solubility of the nanoparticles effectively. It is worth noting that, thickness of the SiO_2_ needs to be determined by multiple experiments. Since too thick or too thin SiO_2_ layers may affect the luminescence intensity of Ln-UCNPs and make the synthesis process more difficult. (Li and Zhang, 2006)

## 4 Ln-UCNPs-based theranostics

The traditional treatment for tumors, chemotherapy and surgical resection, have disadvantages that must be addressed. Several new therapies have attracted recent attention (Atmaca et al., 2021; Kobayashi et al., 2021; Lu et al., 2021; Pedziwiatr-Werbicka et al., 2021; Zhu et al., 2021). PT mainly includes photodynamic therapy and photothermal therapy. In PDT, irradiating a photosensitizer produces reactive oxygen species (ROS) that cause oxidative damage to cells (Yan et al., 2021a). In PTT, light-induced heating damages cancer cells (Shi et al., 2021). Combining photosensitizer or photothermal agent with Ln-UCNPs can achieve PT with near-infrared light.

### 4.1 Ln-UCNPs-based drug delivery

Drug delivery is an important part of cancer treatment. Generally, Ln-UCNPs can be used as a vehicle for drug delivery to achieve a specific therapy. Mechanisms for drug delivery using Ln-UCNPs include passive, active, and physical targeting. In the passive targeting strategy, drugs are combined with Ln-UCNPs to achieve targeted therapy with the help of the enhanced permeability and retention (EPR) effect. In the active targeting strategy, drugs are combined with Ln-UCNPs, and the interaction between a ligand and receptor, or antigen and antibody, is used to specifically recognize cells to achieve the targeted delivery of drugs. Chen et al. (2017) designed Tm^3+^-doped UCNPs and combined them with light-activated Ru complexes, so that enzyme inhibitors can be released by NIR excitation. In the physical targeting strategy, designer nanoparticles can be used to release drugs in specific locations by external environmental stimulus, such as changes in pH or temperature, photoexcitation, or magnetic targeting. Lin and coworkers designed Ln-UCNPs doped with Yb^3+^ and Tm^3+^ with additional modification of hydrazine monohydrate on the surface. In an acidic environment, the hydrogen bonds in the nanoparticles dissociate, enabling drug release (Yang et al., 2014). Mykhaylyk et al. (2001) designed magnetic nanoparticles that can deliver drugs to penetrate the blood-brain barrier and enter the mouse brain (Mykhaylyk et al., 2001). De et al. designed a polyelectrolyte complex based on Yb^3+^ and Er^3+^ to deliver pharmaceutical grade protein (De et al., 2022). The modified nanosystem overcame the problem of protein aggregation in the cell membrane and protected the protein drugs from destruction by proteases and the action of heat. At the same time, the nanoscale drug carrier system responded to the 980-nm NIR light, and realized the imaging of the protein delivery process.

### 4.2 Ln-UCNPs-based phototherapy

PDT has been clinically applied as a supplement to traditional therapy. Porphyrin is the first photosensitizer approved for this purpose (Tian et al., 2020), and mTHPC (meso-tetra hydroxy phenyl chlorin), a second generation photosensitizer, has shown many excellent characteristics in photodynamic therapy (Pinto et al., 2021; Yakavets et al., 2021; Yuan et al., 2021). In addition, chlorin dihydrogen E6 (Chlorin e6, Ce6), ZnPc and other photosensitizers have been widely used in scientific research although they have not been clinically approved (Chen et al., 2021a; Zhang et al., 2021a; Chen et al., 2021b; Hu et al., 2021; Lee et al., 2021; Shen et al., 2021; Zheng et al., 2021). Most photosensitizers are hydrophobic and easily aggregate in solution, which introduces practical difficulties (Liu et al., 2021a; Lin et al., 2021; Wei et al., 2021; Wu et al., 2021). Compared with PDT, there are less examples of the clinical application of PTT for tumor treatment. ICG is a dye that has been widely used in clinical diagnosis (Lutken et al., 2021; Teng et al., 2021). It can absorb in the near-infrared (600–900 nm) which enhances tissue penetration. In addition to its use in imaging and diagnosis, ICG has high photothermal conversion efficiency for photothermal therapy which is helpful for follow-up treatment of tumors (Ravichandran et al., 2020; Zhang et al., 2021b).

Currently, most of the photothermal agents are still applied in preclinical or early clinical research due to the fact that photothermal reaction cannot not completely eradicate a tumor; furthermore, there are additional risks due to overheating caused by laser ablation (Gao et al., 2020; Guo et al., 2021; Li et al., 2021; Pang et al., 2021; Zhao et al., 2021).

Ln-UCNPs are candidates for phototherapy due to their low toxicity and ability to be excited by near-infrared light. Ln-UCNPs are often used to deliver other molecules or add modifiers, for which the ability to upconvert expands the range of usable therapeutic agents making it more suitable for cancer phototherapy. In most cases, PDT and PTT effects exist simultaneously (Yang et al., 2021a; Bian et al., 2021; Yang et al., 2021b).

**FIGURE 5:**
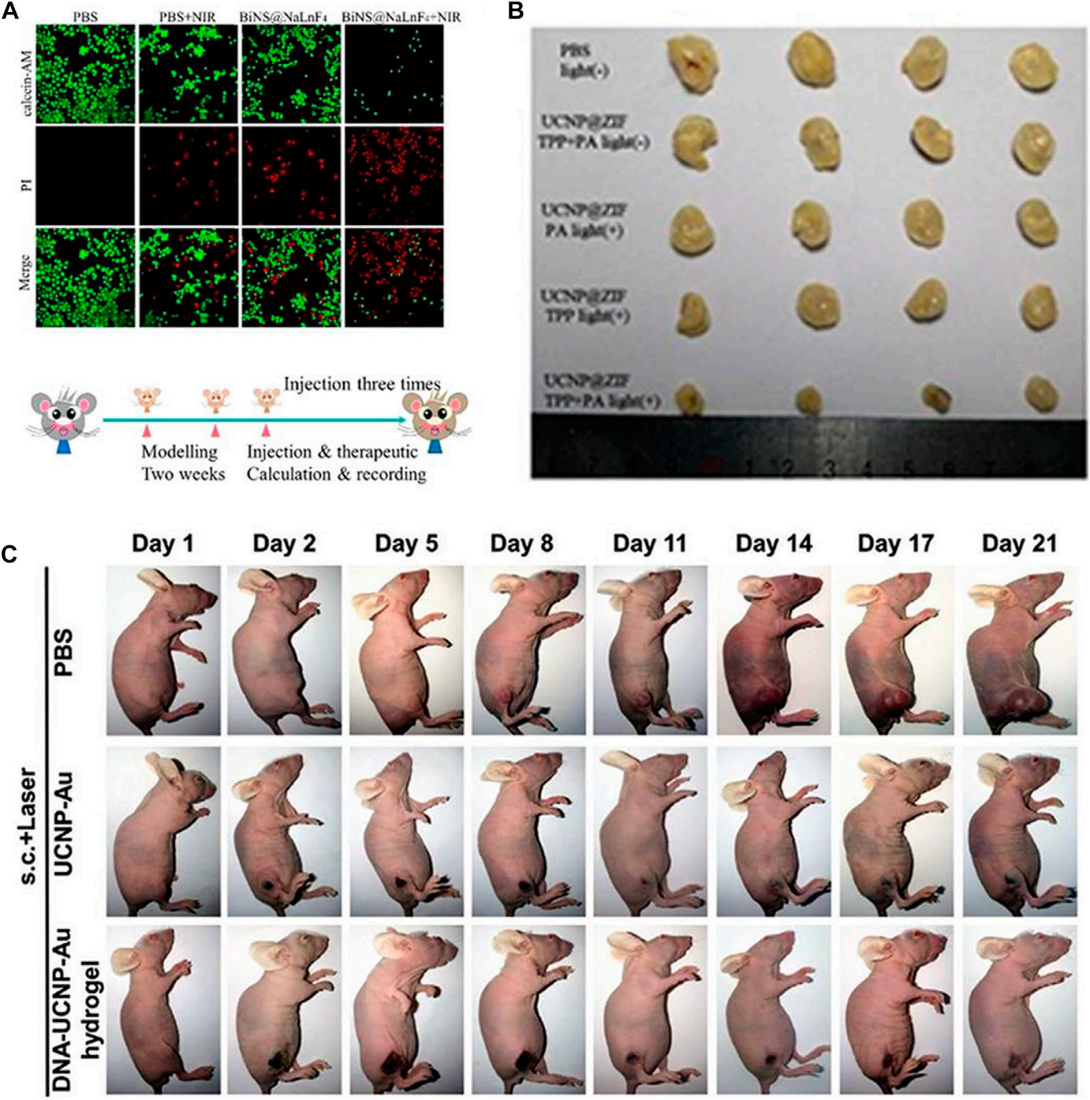
**(A)** *In vitro* PTT confocal images of ECA109 cells treated with PBS and BiNS@NaLnF4, with or without NIR irradiation. [Reprinted with permission from Ref. (Ma et al., 2021a) Copyright 2021: Royal Society of Chemistry]. **(B)** Images of tumor after treatment. [Reprinted with permission from Ref. (Wang et al., 2020) Copyright 2020: Royal Society of Chemistry]. **(C)** Photographs of female BALB/c nude mice bearing T24 tumors when treated with DNA–Ln-UCNPs-Au hydrogel, Ln-UCNPs-Au, and PBS samples over a period of 21 days after NIR irradiation in 3 min (1 W cm^−2^); s.c., subcutaneous injection. [Reprinted with permission from Ref. (Liu et al., 2020) Copyright 2020: John Wiley & Sons, Inc.].

Generally, Ln-UCNPs is used in phototherapy to convert near infrared into visible light to trigger a photodynamic response. Guang and co-workers synthesized BiNS@NaLnF_4_(Ln = Gd) using a solvothermal method (Figure 5A). Specifically, NaGdF4 was loaded onto Bi ultra-thin nanosheets, and up-conversion was observed under 980 nm laser irradiation to stimulate ROS generation. NaLnF_4_ has a light-to-heat conversion efficiency of 35.3% (Ma et al., 2021a). Ln-UCNPs can also be used as a light energy converter to participate in the reconstruction of a tumor’s microenvironment (Figure 5B). The emitted light of a Ln-UCNPs can activate a photoacid to release H^+^, which reduced the pH value of the tumor environment and improved the release of ROS in the cell in response to acid environment (Wang et al., 2020). Liu et al. (2020) synthesized an injectable DNA-Ln-UCNPs-Au hydrogel, which enhanced the aggregation of Ln-UCNPs-Au through electrostatically complexed DNA strands and achieved a photothermal conversion efficiency of 42.7% (Figure 5C). In *in vivo* experiments, DNA-Ln-UCNPs-Au showed good stability and anti-cancer activity. In these preclinical studies, Ln-UCNPs have played an important role for tumor treatment. Since the excitation wavelength is in near-infrared band, phototherapy based on Ln-UCNPs can avoid the self-absorption problem of biological tissues and increase the penetration depth.

### 4.3 Ln-UCNPs-based phototherapy with chemotherapy

**FIGURE 6:**
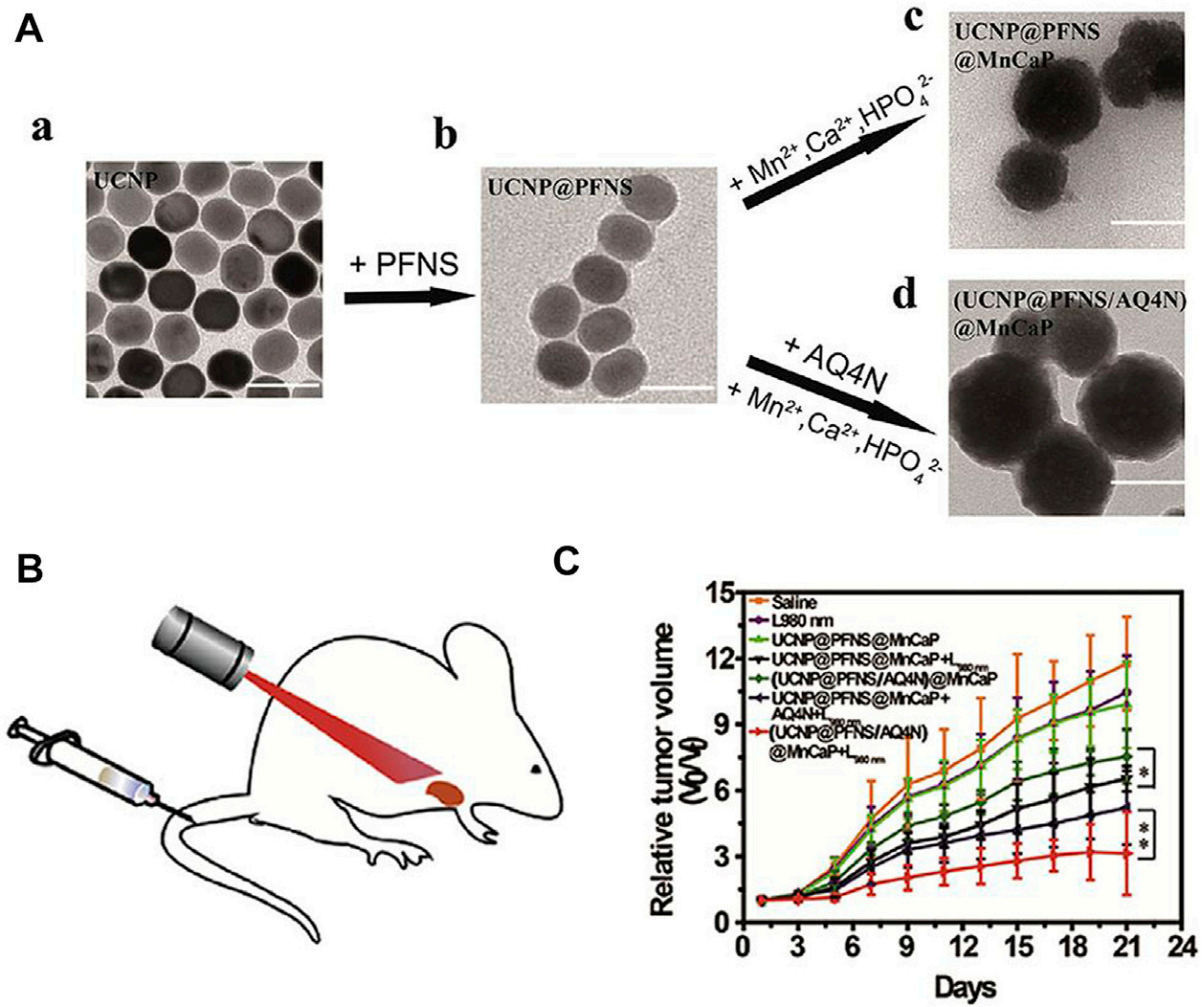
**(A)** Transmission electron microscope (TEM) images of Ln-UCNPs (a), Ln-UCNPs@PFNS (b), Ln-UCNPs@PFNS@MnCaP (c) and (Ln-UCNPs@PFNS/AQ4N) @MnCaP (d). **(B)** Schematic of the treatment of mice with intravenously injected (Ln-UCNPs@PFNS/AQ4N)@MnCaP and treatment by illumination. **(C)** The tumor growth curves for different treatments. Error bars indicate SD (*n* = 6). [Reprinted with permission from Ref. (Ji et al., 2019) Copyright 2019: Elsevier].

Chemotherapy can address the shortcomings of phototherapy, while phototherapy can more accurately target tumors and can be used to visually monitor drug release when combined with bioimaging (Andre et al., 2020; Birtle et al., 2020; Zheng and Xu, 2020; Yan et al., 2021b). A Ln-UCNPs-based multifunctional platform, Ln-UCNPs@PDA@Cy3-pep, that has treatment and real-time monitoring functions was reported (Liu et al., 2021b). The Ln-UCNPs comprised the core, while the chemotherapeutic drug staurosporine (STS) was loaded on the polydopamine (PDA) shell. The complex was shown to be effective for both photothermal therapy and chemotherapy under near infrared light irradiation. The quenching and restoration of Cy3 fluorescence was used to monitor the anti-cancer efficiency of the complex in real time (Liu et al., 2021b). Ju and coworkers reported a combination of chemical-photodynamic therapy based on a dual emitting Ln-UCNPs that was functionalized with a photosensitizer and the prodrug camptothecin. The surface was modified to enhance water solubility and targeting. Near-infrared excitation resulted in ultraviolet emission that activated the toxicity of CPT, and additional blue emission activated the photosensitizer to produce singlet oxygen to kill cancer cells (He et al., 2021). Huang and coworkers modified the photosensitizer to the surface of Ln-UCNPs to obtain Ln-UCNPs@PFNS. AQ4N, a hypoxia-activated prodrug that displays high toxicity selectively to hypoxic environment, was added to the pH-sensitive surface coating for chemotherapy (Figure 6). The surface coating decomposed at the tumor site due to the acidic environment, resulting in exposure of AQ4N and the photosensitizer. Under near-infrared excitation the photosensitizer generated ROS and aggravated the hypoxia at the tumor site which enhanced the therapeutic effect of hypoxia-activated AQ4N (Ji et al., 2019). It is promising to actively explore therapies that can assist chemotherapy given its role as the dominant cancer treatment. It is foreseeable that the structure and optical properties of Ln-UCNPs will add value in chemotherapeutic approaches to treat cancer more effectively (Liang et al., 2020b).

### 4.4 Ln-UCNPs-based phototherapy with immunotherapy

**FIGURE 7:**
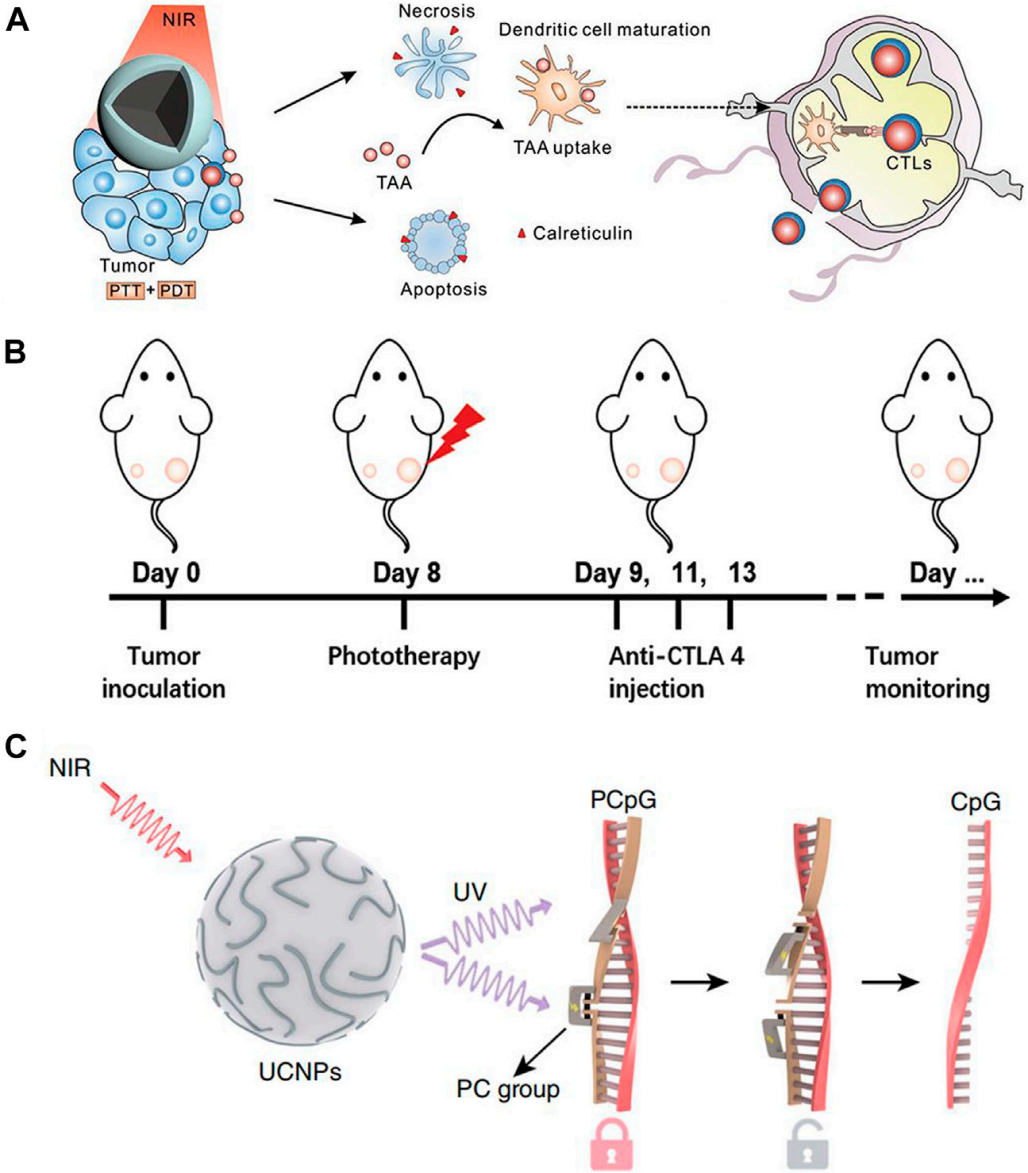
**(A)** Scheme of synergistic phototherapy for augmentation of antitumor immunity. Upon laser irradiation, nanoparticles ablate the primary tumor through phototherapy. (Reprinted with permission from Ref. (Yan et al., 2019) Copyright 2019: John Wiley & Sons, Inc.). **(B)** Schematic depiction of the experimental approach for the evaluation of the abscopal effects induced by Ln-UCNPs/ICG/RB-mal based phototherapy. (Reprinted with permission from Ref. (Wang et al., 2019a) Copyright 2019: John Wiley & Sons, Inc.). **(C)** Schematic of the design of a photoactivatable immunodevice through the integration of Ln-UCNPs with the UV light-responsive photoactivatable CpG (PCpG). Ln-UCNPs act as transducer to upconvert NIR light into UV light locally, thus liberate CpG oligonucleotides (ODN) from PCpG to achieve refined temporal control on its immunoactivity. [Reprinted with permission from Ref. (Chu et al., 2019) Copyright 2019: Nature Publishing Group].

In recent years, immunotherapy has attracted attention due to the targeting capability and increased safety. Tumor immunotherapy does not reply on external stimuli, rather, it activates the immune response and cultivates immune cells to attack cancer cells (Tan et al., 2020a). Unlike traditional therapies, cancer cells can be removed continuously and quickly. Due to the efficacious anti-cancer effects of anti-CTLA-4 and anti-PD-1 antibodies, immunotherapy has the potential to revolutionize tumor treatment (Kamata-Sakurai et al., 2021; Tay et al., 2021). Many studies have demonstrated the potential for immunotherapy to improve the survival rate of patients and improving quality of life (Borcoman et al., 2019; Wallis et al., 2019; Mpekris et al., 2020). The combination of Ln-UCNPs-based phototherapy and immunotherapy may augment each other’s therapeutic effects. Liu et al. prepared a core-shell structure with a core PDA, a NaGdF_4_:Yb/Er shell, and modified it with the photosensitizer Ce6 (Figure 7A). When irradiated with a 980 nm laser, the PDA component of the PDA@Ln-UCNPs-PEG/Ce6 acted as a photothermal agent while the Ce6 functioned as a photodynamic therapy agent. Phototherapy stimulated the body to produce an immune response, activated T lymphocytes and T memory cells, and helped to inhibit tumor metastasis and recurrence. However, it did not protect the mice from death threats caused by cancer. When combined with the immunosuppressant PD-1, the survival rate of mice was significantly improved to 77.8%, demonstrating the advantages of immunotherapy in long-term survival (Yan et al., 2019). Similarly, Chen and co-authors constructed a Ln-UCNPs/ICG/rose bengal (RB-mal) system (Figure 7B) that triggered enhanced phototherapy by ICG under near-infrared irradiation. Tumor-derived protein antigen was captured by the nano-platform and kept in place to trigger a systemic anti-tumor immune response. Experiments in mice proved that Ln-UCNPs/ICG/RB-mal can not only cure tumors *in situ*, but also inhibits the growth of distant tumors, illustrating the generation of a tumor-specific immune response (Wang et al., 2019a). In addition, Ln-UCNPs can stimulate the immune response. Li and co-workers combined Ln-UCNPs with PCpG for immunotherapy (Figure 7C). The core-shell nanoparticles NaGdF_4_:70%Yb, 1%Tm@NaGdF_4_ emitted at 313, 363, 453 and 478 nm under 980 nm laser excitation. The UV light activated PCpG to release the immunotherapy agent CpG. In mouse experiments, it was worth noting that the CpG/UCs conjugate proved more effective compared to PCpG/UCs + NIR; however, the former caused severe systemic toxicity which reveals the advantages of using Ln-UCNPs in this process (Chu et al., 2019).

When combining phototherapy with immunotherapy, both the near-infrared irradiation and phototherapy reagents elicit the body’s immune response (Hou et al., 2018; Dai et al., 2020; Sun et al., 2020). However, the process is not clear, and as such exploring complex systems that can cause an immune response is still an urgent issue to be solved.

## 5 Ln-UCNPs-based bioimaging

Recent research in biological imaging has centered on the design of new optical molecular probes (Yao et al., 2020).

Since biological tissue is a high-scattering medium, current imaging technology has disadvantages such as low penetration depth, interference from biological autofluorescence, and may cause tissue damage which greatly limits the application of biological imaging (Jia et al., 2020). Ln-UCNPs have a strong anti-Stokes shift, and the ability to absorb near-infrared light helps avoid biological background fluorescence interference (Dhal et al., 2020). Furthermore, near-infrared light has a deep tissue penetration depth. However, Ln-UCNPs cannot be directly applied to biological imaging due to fluorescence quenching, hydrophobicity, biological toxicity and other problems. Addressing these issues requires modification to their structure (Zhou et al., 2020).

### 5.1 Ln-UCNPs-based fluorescence imaging

Fluorescence imaging generally uses photoluminescence probes, for which Ln-UCNPs are excellent candidates as their application does not result in tissue damage (Hu et al., 2020; Zhang et al., 2020). Recently, Hao and co-workers synthesized polyacrylic acid-modified NaLnF4: 40Gd/20Yb/2Er, (Ln = Y, Yb, Lu, PAA-Ln-NRs). PAA-Ln-NRs with bright NIR II emission were injected into the tail vein of mice, and under excitation from a 980 nm laser they achieved high-precision imaging of small tumors and metastases that enabled early cancer diagnosis (Wu et al., 2019). Liu et al. synthesized dye-sensitized Ln-UCNPs with a reduced propensity for aggregation in water through hydrophobic coordination. Issues with the quenching of Ln-UCNPs luminescence in water was addressed by adjusting the dye absorption band (Liang et al., 2020a). Chen et al. designed an ingenious NaYF_4_@NaYbF_4_@NaYF_4_:Yb^3+^/Tm^3+^@NaYF_4_ structure for multi-channel fluorescence imaging. After intravenous and subcutaneous injection of the Ln-UCNPs, clear *in vivo* color imaging was obtained (Li et al., 2020b). The Fuyou group (Qiu et al., 2017; Liu et al., 2018b; Park et al., 2018) recently synthesized a rare-earth probe with NIR-II imaging capability. Ce^3+^ doping significantly increased the intensity of NIR-II luminescence, resulting a higher resolution in *in vivo* tumor imaging (Cao et al., 2020).

There are some interesting new studies for Ln-UCNPs outside *in vivo* imaging. Giri et al. coated Ln-UCNPs with oleylamine to obtain OA-Ln-UCNPs, and then further processed it to make a Ln-UCNPs oleogel with better skin permeability. The loading of Ln-UCNPs in the oleogels was varied to study the relationship between concentration and fluorescence intensity. For *in vitro* experiments, the Ln-UCNPs oleogel could penetrate the skin more deeply than free OA-Ln-UCNPs and be used as a skin tissue imaging agent under near-infrared excitation. Xu and co-workers reported the application of Ln-UCNPs in plant cell imaging, whereby LiErF4:1%Tm^3+^@LiYF4 with a core-shell structure could enter the cell membrane easily and emit bright red light (Qiao et al., 2021).

### 5.2 Ln-UCNPs-based computed tomography

Computed tomography (CT) is based on the difference in the absorption and transmittance of X-rays among various tissues of the human body. The data is scanned by an instrument and processed to determine the pathological condition (Pfeiffer, 2018). Lanthanides have high X-ray attenuation coefficients (Issa et al., 2018), for example, the atomic number and electron density of Lu and Yb are greater than iodine which is currently used in CT. Du et al. reported the synthesis of core-shell nanoparticles NaYF_4_:Yb/Er@NaLuF_4_:Nd/Yb@NaLuF_4_ and surface modification of chitosan. Next, Ag_2_Se was grown *in situ* to form NaYF_4_:Yb/Er@NaLuF_4_:Nd/Yb@NaLuF_4_@CS@Ag_2_Se. *In vivo* imaging experiments with these agents demonstrated potential for clinical CT (Du et al., 2020). Bismuth doping of materials significantly improves CT imaging as Bi has better X-ray attenuation ability compared to lanthanides. You et al. reported the synthesis of a porous BiF3:Yb, Er nanomaterial that generated an *in vivo* CT signal as demonstrated with concentration dependence. The CT value curve showed that the Hounsfield unit (HU) value of the sample was higher than observed in commercial Iohexol (Zhao et al., 2020). Gao and co-workers reported the synthesis of BaYF5 with different bismuth doping ratios: Yb, Er, Bi-x (x = 0–3). Under the excitation of a 980 nm laser, the up-conversion luminescence was enhanced with increasing Bi content, reaching a maximum at x = 2.5. Then Ln-UCNPs was coated with citrate for biological imaging. Both *in vitro* and *in vivo* experiments demonstrated that the Ln-UCNPs can be successfully used in X-ray CT imaging by accumulating at the tumor site (Luo et al., 2020).

### 5.3 Ln-UCNPs-based magnetic resonance imaging

**FIGURE 8:**
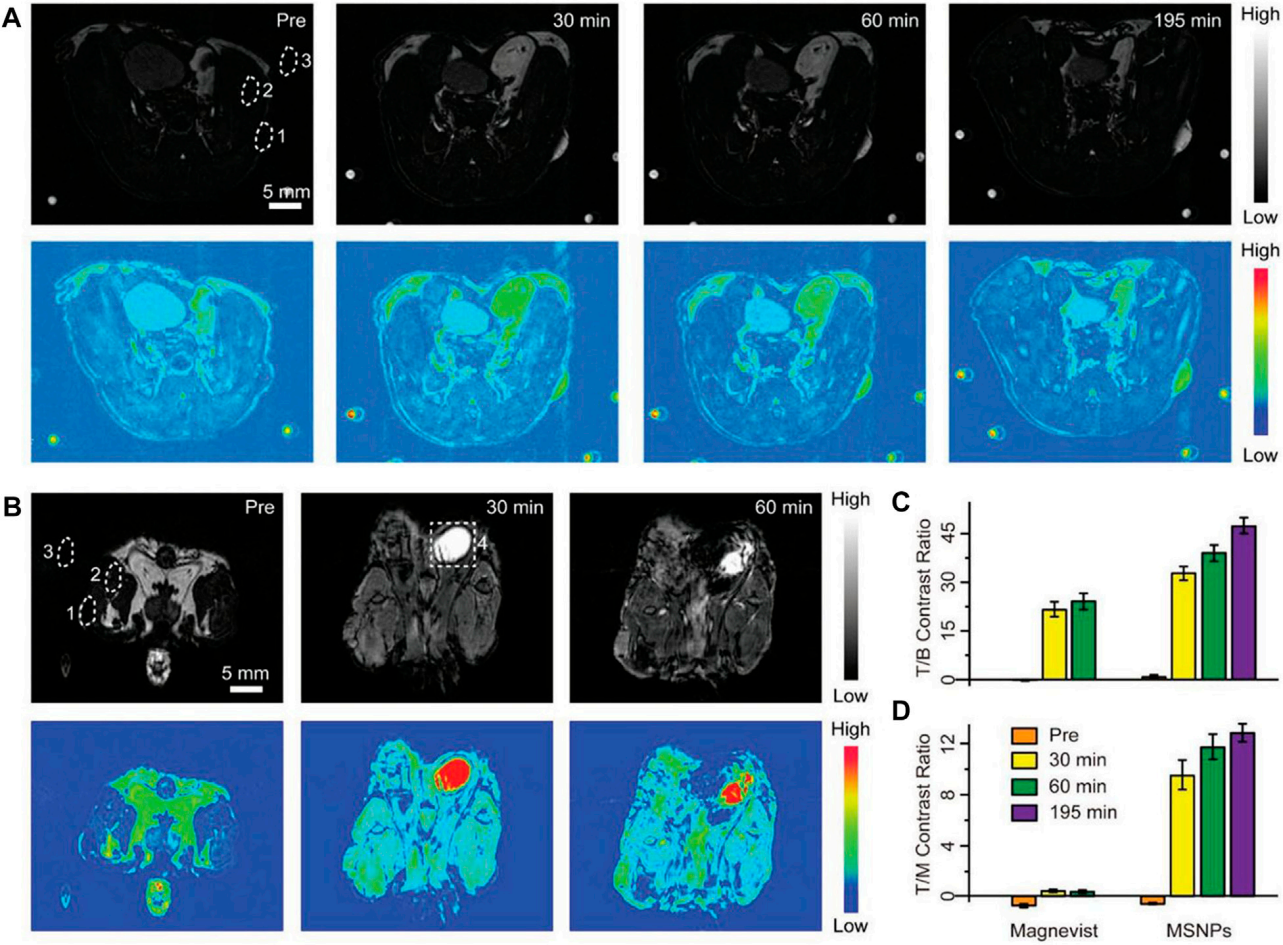
Positive contrast enhancement evaluation *in vivo*. **(A, B)** T1-weighted MRI and corresponding pseudocolor images of tumor-bearing mice after intravenous injection of cell membrane coated-BSNPs (MSNPs) **(A)** and Magnevist **(B)** with the same dosage (2.5 μmol of Gd^3+^ for each mouse). Images were captured before and at different time points after the administration of contrast agents. The time points were collected at the midpoint of the time interval during each imaging acquisition. The dotted circles represent the regions of interest: 1) tumor, 2) muscle, 3) background, and 4) bladder. Scale bars are 5 mm for all images. The small spots on the corners are from the circulation apparatus in the MRI scanner. **(C, D)** Tumor-to-background (T/B) and tumor-to-muscle (T/M) contrast ratios based on the corresponding MRI images. Values represented as means ± s.d. (*n* = 3). [Reprinted with permission from Ref. (Yi et al., 2019) Copyright 2019: John Wiley & Sons, Inc.].

Magnetic resonance imaging is a technique in which radio frequency pulses are applied to generate structural information (Tirotta et al., 2015; Clough et al., 2019). The signal is generated from relaxation of nuclei from the excited state to equilibrium over the relaxation time. There are two relaxation times, labeled T1 and T2, where T1 is the spin-lattice or longitudinal relaxation time and T2 is the spin-spin or transverse relaxation time. Therefore, MRI is divided into T1 MRI and T2 MRI. The lanthanide gadolinium is the main contrast agent for T1 MRI due to its paramagnetic properties (Li et al., 2014; Yang et al., 2016; Tse et al., 2019). Liu et al. reported a T1-weighted nanocontrast agent with smart MRI switch based on NaGdF_4_. Ultra-small NaGdF_4_ and pH-sensitive CaCO_3_ generated self-assembled bare self-assembled nanoparticles (BSNPs) in organic solvents (Figure 8). The addition of a tumor targeting coating on the surface effectively guided the BNSPs to the tumor location, and then CaCO_3_ reacted with the acidic environment to release NaGdF_4_ to achieve MRI with 60 times higher contrast compared to the commercial contrast agent Magnevist (Yi et al., 2019). Superparamagnetic Fe_3_O_4_ nanoparticles combined with Ln-UCNPs are often used in T2 MRI. Li et al. synthesized Fe_3_O_4_@NaGdF_4_:Yb:Er-HMME with a core-shell structure, which was used for both treatment and imaging. The super paramagnetism of the sample was first confirmed, and its T2-weighted MRI performance was studied. Compared with the commercial T2 contrast agent Feridex, the samples performed better, and were effective for use in *in vivo* T2 MRI (Wang et al., 2019b).

### 5.4 Other imaging based on Ln-UCNPs

Single-Photon Emission Computed Tomography (SPECT) and Positron Emission Tomography (PET) are two techniques in nuclear medicine. PET is the only imaging technique that can interrogate biomolecular metabolism, receptors, and neuromediator activity *in vivo*. Generally, substances necessary for metabolism, such as glucose, are labeled with short-lived radionuclides such as ^18^F and ^11^C. After injection into the human body, metabolic activities are reflected by the accumulation of the radiolabeled probes to enable diagnosis. Fang et al. (2020) used cancer cell membrane-modified NaGdF_4_:Yb,Tm@NaGdF_4_ for UCL/MRI/PET trimodal tumor imaging and successfully differentiated triple-negative breast cancer subtypes MDA-MB-231 and MCF-7. Sun et al. (2011) used an efficient strategy for labeling Ln-UCNPs with ^18^F, and successfully applied the materials in sentinel lymph nodes mapping with PET imaging and detection. The materials were realized by the reaction between rare Earth cations and fluoride ions. The process was simple, fast, and efficient as no organic reagents were applied.

The principle of SPECT is to label probes with short half-life nuclides and inject them intravenously. The nuclides emit γ-rays through decay, and the γ-rays are then converted into electrical signals and input into a computer for tomography reconstruction. A cross-section or three-dimensional image reflecting the physiological condition of organs in the human body can be obtained. Li et al. (2020b) prepared radioactive PEG-modified NaYF_4_:Yb, Er, Sm. These 10-nm nanoparticles were detected in kidney and urine by SPECT imaging and gamma counter analysis, confirming the potential for these probes as a biodistribution markers (Cao et al., 2013). Ma et al. (2021b) prepared a platelet membrane-coated nanostructure (PM-PAAO-Ln-UCNPs) containing Ln-UCNPs and Ce6 photosensitizer (PAAO = poly(noctylamine) acrylate), and injected them into mice models. SPECT/CT dual-mode imaging was performed and the precise location of an atherosclerotic plaque was demonstrated.

## 6 Ln-UCNPs-based biosensing

The unique optical properties of Ln-UCNPs make them effective for biological analysis and sensing. First, they are easily incorporated into a fluorescence analysis scheme due to their robust emission properties and high chemical stability. Second, the near-infrared excitation of Ln-UCNPs can effectively avoids autofluorescence of biological tissues. The use of Ln-UCNPs as fluorescent sensors has been extensively studied (Laurenti et al., 2016; Gerelkhuu et al., 2019; Yuan et al., 2019; Su et al., 2020; Gu et al., 2021). This section introduces the application of Ln-UCNPs-based pH and temperature sensors, as well as gas and ion sensors and some other platforms. And the typical cases of Ln-UCNPs for sensing are shown in Table 2.

**TABLE 2:** Typical cases of Ln-UCNPs for sensing.

No	Sensor system	Excitation (nm)	Emission	Sensor type	Recent advances	References
1	NaYF_4_:Yb,Tm-T (Thymine)	980	475 nm	ECL sensor	In the presence of Hg^2+^, the T monolayer-modified Au electrode (AuE/T) absorbed Hg^2+^and T-Ln-UCNPs by T-Hg^2+^-T matching. Surface-tethered T-Ln-UCNPs further recruited more Hg^2+^, as well as T-Ln-UCNPs, thus forming a Ln-UCNPs-T-Hg^2+^-T-Ln-UCNPs reticular architecture on the surface of the electrode	Dhal et al. (2020)
2	ssDNA-NaYF_4_:Yb,Er@SiO_2_	980	549, 654 nm	miRNA sensor	In the absence of complementary miRNA sequences, the ssDNA functionalized particles interact with the GQD leading to an enhancement of the up-conversion emission. In the presence of the target miRNA sequences, the hybridization process yields dsDNA on the surface of the Ln-UCNPs that hinders the interaction with GQD and reduces the up-conversion fluorescence enhancement.	Zhou et al. (2020)
3	NaLuGdF_4_:Yb,Er-Fe^3+^, C u^2+^, and Li^+^	980	543 nm	CAs sensor	It was found that catecholamines could be more effectively detected in the presence of Ln-UCNPs-Fe^3+^, whereas, dopamine and epinephrine were detected selectively using Ln-UCNPs-Li^+^ and Ln-UCNPs-Cu^2+^ sensors.	Hu et al. (2020)
4	APTEs-NaYF4:Yb,Er-Pt	980	543 nm	TNT sensor	The Janus capsule motors were fabricated by layer-by-layer assembly of Ln-UCNPs-functionalized polyelectrolyte microcapsules, followed by sputtering of a platinum layer onto one half of the capsule.	Zhang et al. (2020)
5	NaGdF_4_:Yb,Er@SiO_2_–Spiropyran	980	540 nm	HIS fluorescent nanosensor	HIS could specifically bind to SP, which could cause the isomerization of SP. His will lead to fluorescence quenching of the sensor based on inner filter effects (IFE)	Wu et al. (2019)
6	NaYF_4_:Yb,Er-pHrodo	980	550, 590 nm	pH sensor	A nanosensor based on up-conversion resonance energy transfer (UC-RET) between an upconverting nanoparticle (Ln-UCNPs) and a fluorogenic pH-dependent dye PHrodo ™ Red that was covalently bound to the aminosilane surface of the nanoparticles. The sensitized fluorescence of the pHrodo™ Red dye increases strongly with decreasing PH.	Liu et al. (2018b)
7	NaYF_4_:Yb,Tm-BODIPY	980	451, 475, 64 8 nm	pH sensor	The blue UCL of NaYF4:Yb^3+^,Tm^3+^Ln-UCNPs excited at 980 nm, that overlaps with the absorption of the pH-sensitive fluorophore, provides reabsorption based excitation of the dye, the spectrally distinguishable green fluorescence of which is switched ON upon protonation, preventing photoinduced electron transfer within the dye moiety, and the pH-inert red UCL act as reference.	Park et al. (2018)
8	NaGdF4:Yb,Er-mOrange	980	540 nm	pH sensor	The Ln-UCNPs-mOrange nanoprobe could be fluorescently imaged with 980 nm excitation, having deep penetration depth, by a fluorescence microscope on a coverslip, or uptaken in a single HeLa cell. Nigericin mediated intracellular pH (3.0, 5.0, and 7.0) could be accurately estimated from the CLSM derived FRET ratio.	Qiu et al. (2017)
9	La_2_MoO_6_:Er	379	555 nm	Temperature Sensor	By means of a fluorescence intensity ratio (FIR) technique, the temperature sensing performances in the temperature range of 303–463 K were investigated based on thermally coupled levels, ^2^H11/2 and ^4^S3/2, of Er^3+^ ions. The sensor sensitivity of Er^3+^-activated La_2_MoO_6_ nanoparticles can be greatly affected by the doping concentration and the maximum sensor sensitivity was determined to be about 0.0097 K^−1^ at 463 K.	Du et al. (2020)
10	NaGdF_4_:1%Tm/49%Yb@NaGdF_4_ :15%Tb/1.5%Eu	980	545 nm	Temperature sensor	The large difference in energy between the emission of Tb^3+^and Eu^3+^made the spectra easier to be detected and calculated based on relative intensity measurement compared to single-ion based systems.	Zhao et al. (2020)
11	NaLuF_4_:Mn,Ln-PNIPAM-Au	980	544, 660,	Temperature sensor	By utilizing red/near-infrared dual emitting NaLuF4:Mn^2+^,Ln^3+^(Ln^3+^ = Yb^3+^, Er^3+^, Tm^3+^) Ln-UCNPs as the energy donor and Au nanoparticles as the acceptor, the temperature resolution of the Ln-UCNPs is significantly increased from 3.1°C to 0.9°C in the physiological temperature range.	Luo et al. (2020)
12	NaY(WO_4_)_2_:Er,Yb	980	552, 655 nm	Temperature sensor	The maximum relative sensitivity (Srel) and absolute sensitivity (Sabs) were determined to be ∼1.2% K^−1^ at 293 K and ∼0.9% K^−1^ at 503 K, respectively. The excellent repeatability of fluorescence intensity ration (FIR) and low temperature uncertainty ΔTmin of ∼0.4 K at 293 K make this optical nanothermometry cover a wide temperature range of 293–503 K.	Li et al. (2014)
13	Bi_2_Ti_2_O_7_:Yb,Ho	980	550,665,750 nm	Temperature sensor	The maximum relative sensitivity is calculated to be 2.44% at 498 K, obtained from the temperature-dependent spectra by recording in the range of 298–498 K. The samples also provide excellent repeatability and chromaticity stability.	Tse et al. (2019)
14	NaYF_4_:Yb,Er	980	525,548 nm	Temperature sensor	The spider silks were drawn directly from Araneus ventricosus and were decorated with core−shell Ln-UCNPs *via* a photophoretic effect. By measuring the fluorescence spectra of the Ln-UCNPs on the spider silks, the membrane temperature of a single breast cancer cell was obtained with absolute and relative sensitivities ranging from 3.3 to 4.5 × 10^−3^K^−1^and 0.2–0.8% K^−1.^	Yang et al. (2016)
15	NaYF_4_:Yb,Er-polystyrene	980	542,657 nm	Gas sensor	PS is chosen as a matrix because it displays permeation selectivity for CO_2_ and rejects protons. The luminescence intensities of the Ln-UCNPs at 542 and 657 nm increase with increasing concentration of CO_2_, and the detection limit is 0.11% of CO_2_.	Cao et al. (2013)
16	NaYF_4_:Yb,Tm@PEP	980	474 nm	Fe^3+^ sensor	Limit of detections (LOD) of 0.2 μM and recoveries of 94.5–102.5%, Ln-UCNPs@PEP was shown to have low cytotoxicity and was used for monitoring Fe^3+^ in HeLa cells by fluorescence microscopy.	Gerelkhuu et al. (2019)

### 6.1 Ln-UCNPs-based pH sensors

**FIGURE 9:**
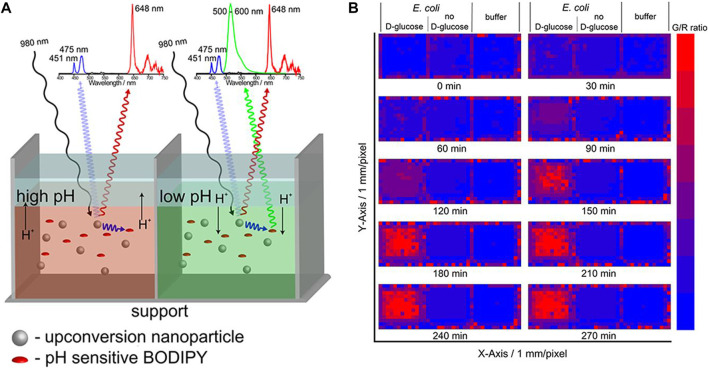
**(A)** Simple self-referenced luminescent pH sensors based on up-conversion nanocrystals and pH-sensitive fluorescent BODIPY dyes. **(B)** Time-dependent changes of the G/R ratio of the pH sensor layers treated with a suspension of E. coli with D-glucose and a suspension of E. coli without D-glucose or pure buffer. [Reprinted with permission from Ref. (Radunz et al., 2019) Copyright 2019: American Chemical Society].

The pH of the cell can reflect its condition; for example, abnormal cells are acidic. Furthermore, as some viruses and germs are most active under acidic conditions, (White et al., 2017) it is important to monitor intracellular pH. Ratiometric (dual color) Ln-UCNPs-based pH ratiometric sensors can be prepared by first analyzing the emission spectrum of Ln-UCNPs to ensure that it has a pH-insensitive component to serve as a reference, and then coupling it to a fluorescent pH indicator. Proper spectral matching results in fluorescence resonance energy transfer that can generate pH-dependent emission. The Schaferling group coupled Ln-UCNPs to a fluorescent pH-dependent dye pHrodoTM Red. The NaYF_4_:Yb^3+^, Er^3+^ material emits at 550 and 660 nm under laser excitation at 980 nm. As the emission peak at 550 nm was found to be invariant to changes in pH, it was used as a reference signal. The dye pHrodoTM Red emitted at 590 nm with an intensity that is dependent on pH. As a result, the combination of the two chromophores created a ratiometric fluorescence probe that displayed a linear response to pH over the range 3–6.7 (Arppe et al., 2014). Likewise Resch-Genger et al. combined NaYF4:Yb^3+^, Tm^3+^ with a pH-sensitive BODIPY dye to create a ratiometric pH sensor (Figure 9). Excitation at 980 nm resulted in 475 nm emission of Tm^3+^ that was absorbed by the dye, while the pH-insensitive 648 nm emission of Tm^3+^ was used as a calibrant against the 528 nm emission of BODIPY. This two-component sensor was successfully applied for pH monitoring in *Escherichia coli* (Radunz et al., 2019). Ln-UCNPs can also be coupled to pH-dependent fluorescent proteins to create ratiometric sensors. Recently, it was reported that Ln-UCNPs were combined with mOrange fluorescent protein. Excitation at 980 nm resulted in UCL green emission by the Ln-UCNPs, which subsequently excited the mOrange fluorescent protein. The ratio of the pH independent Ln-UCNPs emission at 655 nm to the pH- dependent emission of mOrange fluorescent protein was a quantifiable metric that was shown to have good stability and reversibility (Ghosh et al., 2020).

### 6.2 Ln-UCNPs-based temperature sensors

Sensitive, convenient, and biocompatible temperature sensors are needed for biological studies (Tran Quang et al., 2016; Ge et al., 2020). Some rare Earth particles have temperature sensitive luminescence properties, which makes them good candidates for biological thermal sensing (Du et al., 2014; Zheng et al., 2014; Wang et al., 2015; Du and Yu, 2017). Lin et al. synthesized doubly-doped NaLuF4:Mn^2+^, Ln^3+^(Ln^3+^ = Yb^3+^, Er^3+^, Tm^3+^), which have red/NIR dual emission that can be enhanced by the SPR effect of proximal Au nanoparticles. Embedding both these materials into the thermoresponsive polymer poly(N-isopropylacrylamide) (PNIPAM) resulted in a temperature dependent modulation of the Ln-UCNPs and Au nanoparticle distance. The effect was to modulate the red to NIR emission ratio, which was the metric of the temperature-sensitive sensor that had a resolution of 0.9°C (Xiao et al., 2014). Shahzad et al. (2019) co-doped microfibers with LiYF_4_:Yb^3+^/Er^3+^, polymethyl methacrylate, and silver to create temperature sensors. The temperature dependent ratio of the emission intensity at 522 nm and 541 nm of the Ln-UCNPs was quantified over the range of 303–348 K. Doping with Ag was shown to significantly improve the photostability and temperature response of the composite (Shahzad et al., 2019). A study on the temperature sensitivity of ZnO:Yb^3+^/Tm^3+^ has been reported. The Ln-UCNPs was excited using a 980 nm laser to produce blue and red UCL emission. The UCL decreased at lower temperatures in the range of 300°C–780°C. The ratio of the intensities of the blue and red emission was modulated by 2.1% K^−1^ at 293 K (Li et al., 2017a). Zhong et al. synthesized a new type of 8.5 nm × 12.5 nm nanoparticle of NaY(WO4)2:Er^3+^, Yb^3+^ that were shown to be temperature sensitive over the range 293 K–503 K (Lin et al., 2019). In addition to explore the relationship between luminescence of Ln-UCNPs and temperature, researchers have combined various fiber optic probes and temperature-sensitive Ln-UCNPs. Kumar et al. designed Ln-UCNPs coated with polydimethylsiloxane (PDMS) to obtain better fluorescence emission. The pure Ln-UCNPs and Ln-UCNPs-PDMS composite materials were coated on optical fibers for temperature sensing. The sensor coated with composite material displayed a linear response from 295 to 473 K (Kumar et al., 2020). A recent report demonstrated the coating of a temperature-dependent Ln-UCNPs onto the surface of spider silk, a natural optical fiber, to construct a temperature sensor with good biocompatibility. The change in fluorescence intensity of Ln-UCNPs reflected the temperature change of cancer cells. In addition, the sensor successfully detected temperature changes during apoptosis (Gong et al., 2021). Meiling et al. designed a lanthanide nanoscale temperature measurement system that can be used for the diagnosis of *in vivo* inflammation. The structure included an inert core, an active shell and an inert shell. The thermosensitive lanthanide elements were localized in the intermediate shell to shield from interference of the bioactive environment. This ternary structure enabled nanothermometers to continuously measure temperature changes of up to 4 mm depth in biological tissues, with high temperature sensitivity over a physiological temperature range of 10°C–64°C (Tan et al., 2020b). Although Ln-UCNPs are not the only material used for temperature sensors, their use imparts unique advantages. Ln-UCNPs have adjustable excitation wavelengths and can be excited by near-infrared light, making such temperature sensors beneficial for *in vivo* applications. Additionally, Ln-UCNPs have multi-color luminescence, which enables a wide selection of emission for construction of a temperature sensor.

### 6.3 Ln-UCNPs-based gas sensors

Gas molecules are of great significance in many biological processes. For example, O_2_ is important for maintaining metabolism (Mates et al., 2012). CO_2_ can regulate breathing and the acid-base balance in the body. NO regulates cardiovascular function and improves the immunity of white blood cells (Carpenter and Schoenfisch, 2012; Lundberg et al., 2015; Farah et al., 2018). As a result, the sensing of gas molecules can elucidate the internal physiological behaviors of organisms. In 2010, Achatz et al. (2011) reported a CO_2_ gas sensor based on Ln-UCNPs for the first time. In their system bromothymol blue was used as the luminescence intensity modifier of Ln-UCNPs on polystyrene. Specifically, bromothymol blue the absorption of bromothymol blue quenched the emission of the Ln-UCNPs; however, the presence of acidic CO_2_ changed the pH which instigated a color change. Thus, when the CO_2_ concentration increased, the absorption of bromothymol blue was minimized which resulted in higher intensity Ln-UCNPs fluorescence that enabled a quantitative detection of CO_2_ with a detection limit of 0.11% (Ali et al., 2010). The same group created other gas sensors using Ln-UCNPs. They reported the first application of NaYF_4_:Yb, Tm excited by near-infrared light as an O_2_ sensor. First, an iridium (III) oxygen probe, Ln-UCNPs, and ethyl cellulose were dissolved in tetrahydrofuran. The solvent was evaporated to yield a sensor film. The Ln-UCNPs was excited by a 980 nm laser and emitted at 455 nm and 475 nm. These two emission peaks overlapped the absorption of the iridium complex at 468 nm. Therefore, Ln-UCNPs acted as a nanolamp in the sensor. The fact that the iridium complex’s emission at 568 nm is quenched by O_2_ enabled quantitatively oxygen detection (Achatz et al., 2011).

### 6.4 Ln-UCNPs-based ion sensors

Although the content of metal ions in organisms is small, they play an important role in maintaining the acid-base balance of cells and organisms and other metabolic activities, and are components of many complex biological compounds. The luminescence properties of Ln-UCNPs show great advantages for metal ion detection. Many studies have demonstrated that ion sensors based on Ln-UCNPs have high sensitivity and selectivity, the results from which can be visualized for convenient operation. Iron directly participates in the transport and storage of oxygen. It is a component of hemoglobin, myoglobin and cytochrome, and is necessary for many metabolic processes (Abbaspour et al., 2014; Sangkhae and Nemeth, 2017). The Lee group reported that adrenaline-modified NaYF_4_:Yb, Tm was used for intracellular Fe^3+^ detection. PEP can complex with Fe^3+^, and the energy transfer with the Ln-UCNPs occurred through non-radiative electron transfer and energy return (EBT), resulting in reduced Ln-UCNPs emission at 474 nm. The detection limit of the Fe^3+^ sensor was 0.2 μM over a range of 1–10 μM (Gerelkhuu et al., 2021). The content of zinc in the human body can affect the activity of many enzymes, and as a result Zn^2+^ has an important role in human health (Maret, 2013). Chang et al. synthesized a Zn^2+^ sensor composed of a Ln-UCNPs and a Zn^2+^ sensitive dye.

NaYF_4_:Yb/Tm@NaYF_4_ has a multi-peak emission; the blue component at 475 nm was within in the absorption range of the dye while the 654 nm emission was not affected. When Zn^2+^ was added, the absorption of the dye shifted to 360 nm, which led to an increase in the ratio of I475 nm/I654 nm. The sensor successfully detected Zn^2+^ in a live animal model of zebrafish (Peng et al., 2015).

**FIGURE 10:**
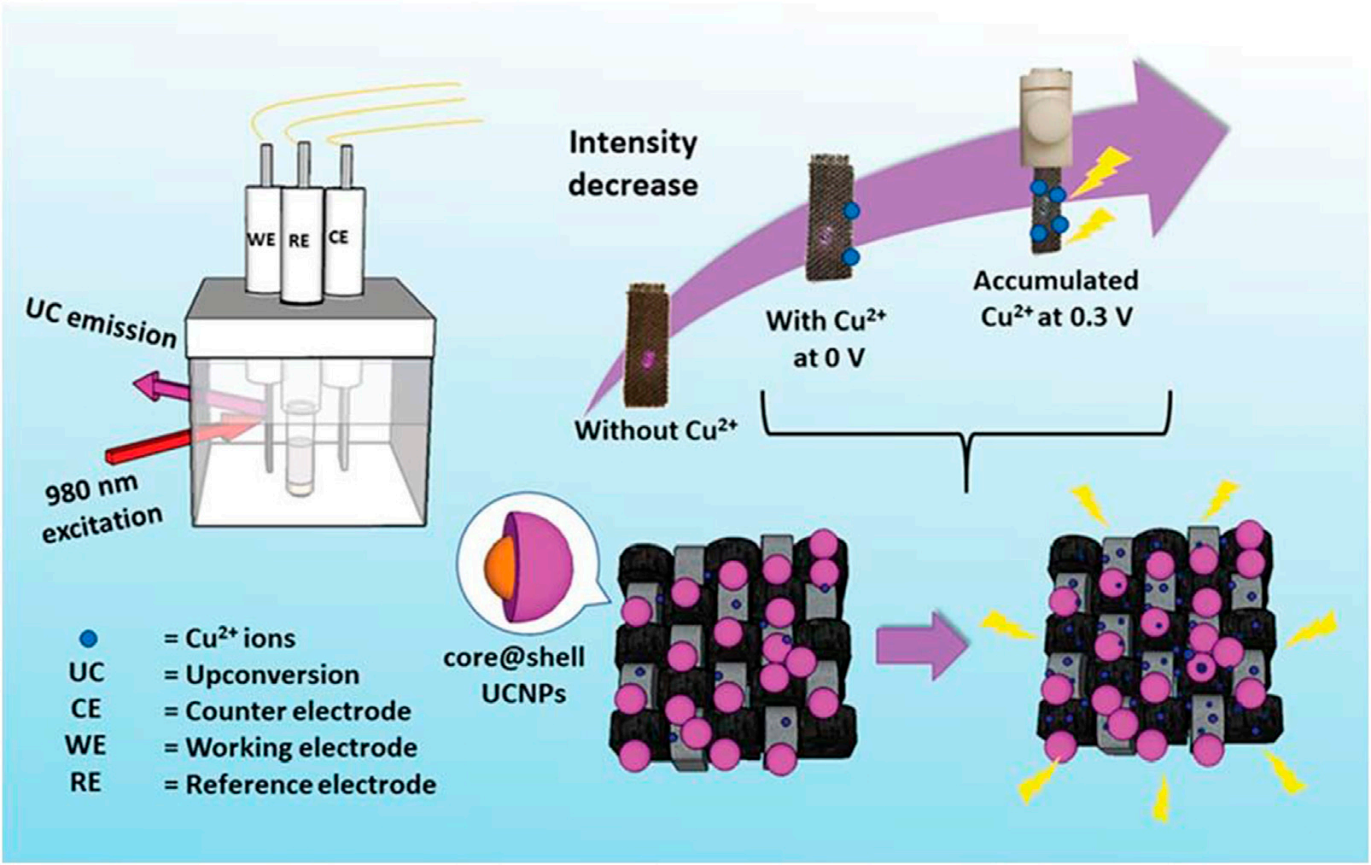
A schematic diagram showing the CFC-Ln-UCNPs probe for Cu^2+^ ion sensing with electrochemical assistance. [Reprinted with permission from Ref. (Wong et al., 2019) Copyright 2019: Royal Society of Chemistry].

Copper is an indispensable component in blood and participates in hematopoietic process and iron metabolism. It was reported that core-shell Ln-UCNPs were electrostatically adsorbed onto flexible carbon fiber cloth (CFC) to create Cu^2+^ sensors (Figure 10). The absorption of Cu^+^ and Cu^2+^ coincided with the emission of Ln-UCNPs, which is a sensing mechanism. It was the first time that electrochemical technology was used to improve the sensing performance. Cu^2+^ accumulated when a voltage of 0.3 eV was applied. CFC reduced the quenching of Ln-UCNPs in water. The synergy of the electrochemical technology and the CFC protective layer resulted in a detection limit of 82 ppb (Wong et al., 2019). Trace fluoride can promote the normal development of teeth and bones, but excessive fluoride causes dental and skeletal fluorosis. Dual-functional gallic acid-Fe(III) modified Ln-UCNPs was reported with a 654 nm emission that was quenched by the Fe-complex. The presence of F^−^ destroyed the Fe-complex, which imparted a sensing mechanism between the UCL and F^−^ ion analyte (Ma et al., 2017).

### 6.5 Other new sensors

**FIGURE 11:**
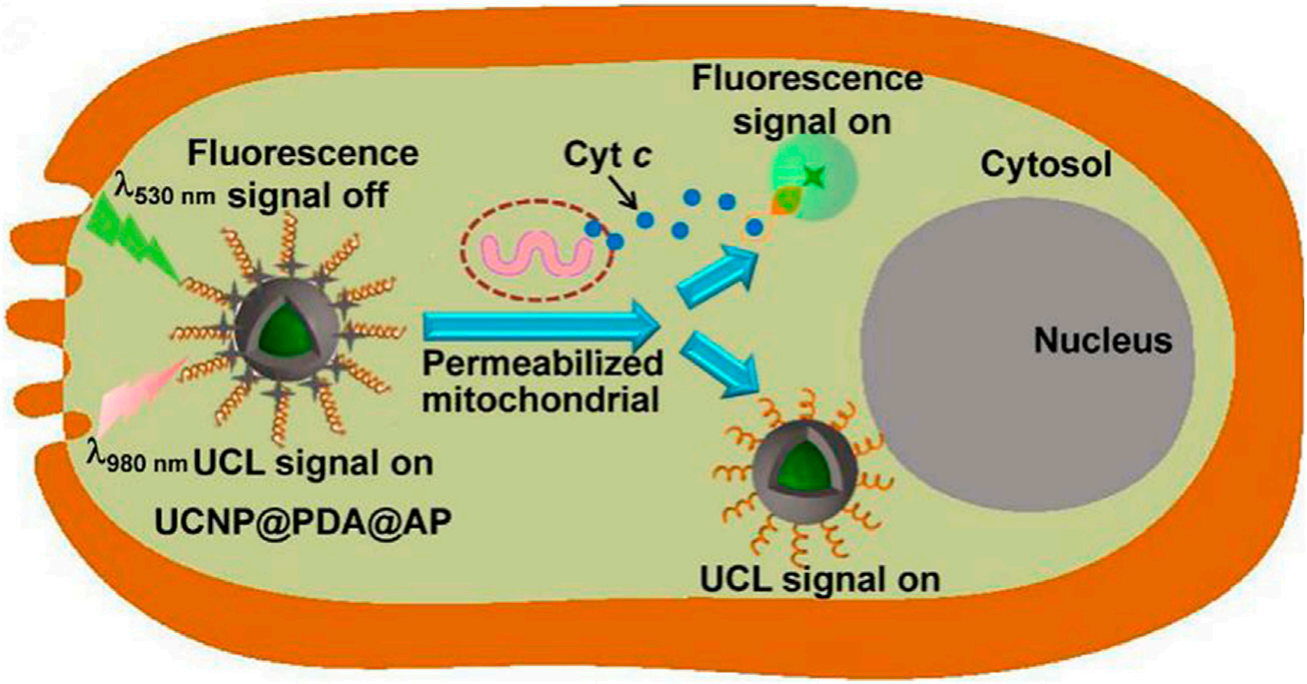
Illustration of the Ln-UCNPs@PDA@AP application for sensing intracellular Cyt c. The illustration is not drawn to scale. [Reprinted with permission from Ref. (Ma et al., 2017) Copyright 2017: Elsevier].

Recently there have been many reports on the use of Ln-UCNPs for the detection of miRNA and other biologically active molecules (Yuan et al., 2015; Li et al., 2016). Trypsin is an activating enzyme that plays an important role in food digestion and has anti-inflammatory and swelling functions (Plattner and Noe, 2015; Wattanasiritham et al., 2016). Guo et al. synthesized Ln-UCNPs-peptide-AuNP for the detection of trypsin and its inhibitors. The peptide, DDDDARC, is sensitive to trypsin. In the initial state, the emission of UCL was quenched by the Au NP due to Förster Resonance Energy Transfer (FRET) energy transfer. After adding trypsin the peptide was cleaved and UCL was restored. The detection limit of the trypsin sensor was 4.15 ng/mL (Wu et al., 2017). Cytochromes are involved in cell redox reactions and take part in cell energy transfer (Zanger and Schwab, 2013). Wang et al. attached a Cy3-labeled aptamer to the surface of a Ln-UCNPs wrapped with PDA (Figure 11). The aptamer responded to cytochrome c to trigger changes in the fluorescence intensity of Cy3, and the internal UCL served as a reference signal. The sensor reflected the level of Cytc through a ratiometric fluorescence signal, with a detection limit of 20 nM over a range of 50 nM to 10 μM (Ma et al., 2017).

Recent studies have demonstrated that a single sensor can be used for the simultaneous detection multiple biomolecules. Xu et al. developed an Au-Au-Ln-UCNPs with an aptamer with sensitivity for both alpha-fetoprotein (AFP) and mucin 1. The sensor generated both Raman and fluorescence signals. Mucin 1 concentration was detected through the change of surface enhanced Raman scattering intensity, and AFP was sensed by the change of fluorescence intensity. The detection limits of both proteins were at an attomolar level (Qu et al., 2017).

Thrombin acts on the last step of the blood coagulation process. It can be used to stop bleeding and is a relevant marker for tumor diagnosis. Prostate-specific antigens are abundantly present in prostate tissue and semen, with extremely high tissue organ specificity, and are currently the first-choice marker for the diagnosis of prostate cancer. Kuang et al. used a Raman signal and fluorescence of Au-Ln-UCNPs in conjunction with aptamers to respond to thrombin and prostate specific antigen (PSA). The signal intensity of SERS was affected by the concentration of thrombin. The fluorescence signal was affected by PSA, and the detection limit was 3.2 × 10^−20^ M (Hao et al., 2017).

## 7 Biological toxicity of Ln-UCNPs

Despite the great potential in applications such as biotherapy, biological imaging and biosensing, the biotoxicity remains a major problem hindering the clinical application of Ln-UCNP. Most of the current studies on *in vivo* toxicity of Ln-UCNPs focus on mice. Ln-UCNPs enter mice in tail vein injection and reach the whole body by blood circulation. Due to the high permeability effect of capillaries to nanoparticles, the liver and spleen become the main aggregate organs of Ln-UCNPs (Li et al., 2017b; Abualrejal et al., 2019; Chen et al., 2019; Guryev et al., 2019; Shan et al., 2020). Chen and co-workers designed targeted contrast agents modified by PEI and FA (Chen et al., 2019). At 24 h and 18 days after the injection, the aggregation of nanoions in liver was higher than that in other tissues. Ln-UCNPs injected into the body can be eliminated by the hepatobiliary and renal metabolic systems. Tian and co-workers (Tian et al., 2019) found that the rate of excretion of Ln-UCNPs is independent of the modified groups. Other studies have shown that the modified group species can influence the toxicity of Ln-UCNPs. Chen and co-workers (Chen et al., 2018) designed a kind of unmodified nanoparticles (NaYF_4_:Er and NaGdF_4_:Yb,Er), which caused mild liver toxicity and nephritis. This can be considered as the unmodified Ln-UCNPs would release rare Earth ions under biological conditions, which will react with the phosphate group of ATP, cause ATP inactivation and tissue damage. Vedunova and co-workers (Vedunova et al., 2016) polymaleic anhydride octadecene, PEI, tetramethyl ammonium hydroxide modified Ln-UCNPs (NaYF_4_:Yb,Tm@NaYF_4_) respectively. The nanoparticles caused the morphological changes of the hippocampal cells, reduction in Ca^2+^ activity and cellular damage. Moreover, the nanoparticle size, concentration, and treatment time can also affect the biotoxicity of Ln-UCNPs (Xu et al., 2016; Chen et al., 2018; Rafique et al., 2018).

## 8 Conclusion and future prospects

In this review, we summarize the latest progress in the synthesis, optimization and application (especially for therapy) of Ln-UCNPs. The high-temperature pyrolysis method and hydrothermal/solvothermal procedure are still the most widespread protocols for their syntheses. In terms of improving up-conversion emission, through algorithm optimization, dye sensitization and other methods, the up-conversion emission intensity can be increased by multiple orders of magnitude. The fluorescent properties of lanthanide upconverting nanoparticles have also advanced the field of biomedicine. Near-infrared excitation addresses the limitations of ultraviolet to visible light excitation, penetrates deeper into tissue, and makes nanotherapeutic materials with light as the main excitation source a strong competitor for clinical drugs. Although lanthanide up-conversion has advanced significantly, there are still a large number of obstacles to address. We posit that the future direction of Ln-UCNPs research in the field of biology consists of: 1) Existing luminescence enhancement strategies are either ineffective or easily quenched when exposed to water. Better results may be obtained starting from the principle of up-conversion luminescence to explore optimization methods. 2) Cancer treatment drugs based on Ln-UCNPs have not been reported clinically, which may be related to long term toxicity in the body. It may be possible to extend the *in vivo* experiment period and minimize other factors that affect drug metabolism to simulate the therapeutic effects to the greatest extent. 3) Although the synthesis technology of Ln-UCNPs is very mature, the products are often hydrophobic. Surface modification for *in vivo* application will improve biocompatibility; however, this process will affect the luminescence efficiency. Finding the balance between biocompatibility and luminescence is a difficult problem. 4) Ln-UCNPs-based biosensors have been developed. However, few studies have evaluated the sensitivity and stability of Ln-UCNPs sensors for *in vivo* sensing. The *in vivo* environment is complex, and how to accurately deliver nanosensors to the detected cellular locations is also a major challenge.

## References

[B1] Abbaspour N. Hurrell R. Kelishadi R. (2014). Review on iron and its importance for human health. J. Res. Med. Sci. 19 (2), 164–174. 24778671 PMC3999603

[B2] Abualrejal M. M. A. Eid K. Tian R. Liu L. Chen H. Abdullah A. M. (2019). Rational synthesis of three-dimensional core-double shell upconversion nanodendrites with ultrabright luminescence for bioimaging application. Chem. Sci. 10 (32), 7591–7599. 10.1039/c9sc01586h 31588310 PMC6761864

[B3] Achatz D. E. Meier R. J. Fischer L. H. Wolfbeis O. S. (2011). Luminescent sensing of oxygen using a quenchable probe and upconverting nanoparticles. Angew. Chemie-International Ed. 50 (1), 274–277. 10.1002/ange.201004902 21031387

[B4] Adachi S. (2018). Photoluminescence properties of Mn4+-activated oxide phosphors for use in white-LED applications: A review. J. Luminescence 202, 263–281. 10.1016/j.jlumin.2018.05.053

[B5] Ali R. Saleh S. M. Meier R. J. Azab H. A. Abdelgawad I. I. Wolfbeis O. S. (2010). Upconverting nanoparticle based optical sensor for carbon dioxide. Sensors Actuators B-Chemical 150 (1), 126–131. 10.1016/j.snb.2010.07.031

[B6] Amouroux B. Roux C. Marty J. D. Pasturel M. Bouchet A. Sliwa M. (2019). Importance of the mixing and high-temperature heating steps in the controlled thermal coprecipitation synthesis of sub-5-nm Na(Gd-Yb)F-4:Tm. Inorg. Chem. 58 (8), 5082–5088. 10.1021/acs.inorgchem.9b00143 30912933

[B7] Andre T. Shiu K. K. Kim T. W. Jensen B. V. Jensen L. H. Punt C. (2020). Pembrolizumab in microsatellite-instability-high advanced colorectal cancer. N. Engl. J. Med. 383 (23), 2207–2218. 10.1056/nejmoa2017699 33264544

[B8] Arppe R. Nareoja T. Nylund S. Mattsson L. Koho S. Rosenholm J. M. (2014). Photon upconversion sensitized nanoprobes for sensing and imaging of pH. Nanoscale 6 (12), 6837–6843. 10.1039/c4nr00461b 24827972

[B9] Atmaca G. Y. Aksel M. Keskin B. Bilgin M. D. Erdogmus A. (2021). The photo-physicochemical properties and *in vitro* sonophotodynamic therapy activity of Di-axially substituted silicon phthalocyanines on PC3 prostate cancer cell line. Dyes Pigments 184, 108760. 10.1016/j.dyepig.2020.108760 36781009

[B10] Bian H. Ma D. Zhang X. Xin K. Yang Y. Peng X. (2021). Tailored engineering of novel xanthonium polymethine dyes for synergetic PDT and PTT triggered by 1064 nm laser toward deep-seated tumors. Small 17 (21), 2100398. 10.1002/smll.202100398 33885221

[B11] Binnemans K. (2015). Interpretation of europium(III) spectra. Coord. Chem. Rev. 295, 1–45. 10.1016/j.ccr.2015.02.015

[B12] Birtle A. Johnson M. Chester J. Jones R. Dolling D. Bryan R. T. (2020). Adjuvant chemotherapy in upper tract urothelial carcinoma (the POUT trial): A phase 3, open-label, randomised controlled trial. Lancet 395 (10232), 1268–1277. 10.1016/s0140-6736(20)30415-3 32145825 PMC7181180

[B13] Bogdan N. Vetrone F. Ozin G. A. Capobianco J. A. (2011). Synthesis of ligand-free colloidally stable water dispersible brightly luminescent lanthanide-doped upconverting nanoparticles. Nano Lett. 11 (2), 835–840. 10.1021/nl1041929 21244089

[B14] Borcoman E. Kanjanapan Y. Champiat S. Kato S. Servois V. Kurzrock R. (2019). Novel patterns of response under immunotherapy. Ann. Oncol. 30 (3), 385–396. 10.1093/annonc/mdz003 30657859

[B15] Cao C. Wu N. Yuan W. Gu Y. Y. Ke J. M. Feng W. (2020). Ln(3+)-doped nanoparticles with enhanced NIR-II luminescence for lighting up blood vessels in mice. Nanoscale 12 (15), 8248–8254. 10.1039/d0nr01098g 32239032

[B16] Cao T. Yang Y. Sun Y. Wu Y. Gao Y. Feng W. (2013). Biodistribution of sub-10 nm PEG-modified radioactive/upconversion nanoparticles. Biomaterials 34 (29), 7127–7134. 10.1016/j.biomaterials.2013.05.028 23796579

[B17] Carpenter A. W. Schoenfisch M. H. (2012). Nitric oxide release: Part II. Therapeutic applications. Chem. Soc. Rev. 41 (10), 3742–3752. 10.1039/c2cs15273h 22362384 PMC3341526

[B18] Chen G. Damasco J. Qiu H. Shao W. Ohulchanskyy T. Y. Valiev R. R. (2015). Energy-cascaded upconversion in an organic dye-sensitized core/shell fluoride nanocrystal. Nano Lett. 15 (11), 7400–7407. 10.1021/acs.nanolett.5b02830 26487489 PMC4915588

[B19] Chen J. Li S. Liu X. Liu S. Xiao C. Zhang Z. (2021). Transforming growth factor-beta blockade modulates tumor mechanical microenvironments for enhanced antitumor efficacy of photodynamic therapy. Nanoscale 13 (22), 9989–10001. 10.1039/d1nr01552d 34076013

[B20] Chen J. P. Shi S. S. Liu G. F. Chen Y. Zheng S. S. Wang X. B. (2018). Potential clinical risk of inflammation and toxicity from rare-earth nanoparticles in mice. Chin. Med. J. Engl. 131 (13), 1591–1597. 10.4103/0366-6999.235105 29941713 PMC6032687

[B21] Chen Q. Ma X. Xie L. Chen W. Xu Z. Song E. (2021). Iron-based nanoparticles for MR imaging-guided ferroptosis in combination with photodynamic therapy to enhance cancer treatment. Nanoscale 13 (9), 4855–4870. 10.1039/d0nr08757b 33624647

[B22] Chen Y. Fei X. Ye C. Qian Q. Ye Z. Xie S. (2019). Acute hepatotoxicity of multimodal targeted imaging contrast agent NaLuF&lt;sub&gt;4&lt;/sub&gt;:Gd,Yb,Er-PEG/PEI-FA in mice. J. Toxicol. Sci. 44 (9), 621–632. 10.2131/jts.44.621 31474743

[B23] Chen Z. J. Thiramanas R. Schwendy M. Xie C. M. Parekh S. H. Mailander V. (2017). Upconversion nanocarriers encapsulated with photoactivatable Ru complexes for near-infrared light-regulated enzyme activity. SMALL 13 (46), 1700997. 10.1002/smll.201700997 29024342

[B24] Chu H. Zhao J. Mi Y. Di Z. Li L. (2019). NIR-light-mediated spatially selective triggering of anti-tumor immunity via upconversion nanoparticle-based immunodevices. Nat. Commun. 10, 2839. 10.1038/s41467-019-10847-0 31253798 PMC6599017

[B25] Clough T. J. Jiang L. Wong K.-L. Long N. J. (2019). Ligand design strategies to increase stability of gadolinium-based magnetic resonance imaging contrast agents. Nat. Commun. 10, 1420. 10.1038/s41467-019-09342-3 30926784 PMC6441101

[B26] Cui Y. Chen B. Qian G. (2014). Lanthanide metal-organic frameworks for luminescent sensing and light-emitting applications. Coord. Chem. Rev. 273, 76–86. 10.1016/j.ccr.2013.10.023

[B27] Dai Y. Yang D. P. Yu D. P. Xie S. H. Wang B. W. Bu J. (2020). Engineering of monodisperse core-shell up-conversion dendritic mesoporous silica nanocomposites with a tunable pore size. Nanoscale 12 (8), 5075–5083. 10.1039/c9nr10813k 32068223

[B28] De R. Song Y. H. Mahata M. K. Lee K. T. (2022). pH-responsive polyelectrolyte complexation on upconversion nanoparticles: a multifunctional nanocarrier for protection, delivery, and 3D-imaging of therapeutic protein. J. Mater Chem. B 10 (18), 3420–3433. 10.1039/d2tb00246a 35389393

[B29] Dhal S. Pal K. Banerjee I. Giri S. (2020). Upconversion nanoparticle incorporated oleogel as probable skin tissue imaging agent. Chem. Eng. J. 379, 122272. 10.1016/j.cej.2019.122272

[B30] Dong H. Sun L.-D. Yan C.-H. (2015). Energy transfer in lanthanide upconversion studies for extended optical applications. Chem. Soc. Rev. 44 (6), 1608–1634. 10.1039/c4cs00188e 25242465

[B31] Du H. Zhang W. Sun J. (2011). Structure and upconversion luminescence properties of BaYF5:Yb3+, Er3+ nanoparticles prepared by different methods. J. Alloys Compd. 509 (7), 3413–3418. 10.1016/j.jallcom.2010.12.101

[B32] Du K. Lei P. Dong L. Zhang M. Gao X. Yao S. (2020). *In situ* decorating of ultrasmall Ag2Se on upconversion nanoparticles as novel nanotheranostic agent for multimodal imaging-guided cancer photothermal therapy. Appl. Mater. Today 18, 100497. 10.1016/j.apmt.2019.100497

[B33] Du P. Luo L. Li W. Yue Q. Chen H. (2014). Optical temperature sensor based on upconversion emission in Er-doped ferroelectric 0.5Ba(Zr0.2Ti0.8)O-3-0.5(Ba0.7Ca0.3)TiO3 ceramic. Appl. Phys. Lett. 104 (15), 152902. 10.1063/1.4871378

[B34] Du P. Luo L. Park H.-K. Yu J. S. (2016). Citric-assisted sol-gel based Er3+/Yb3+-codoped Na0.5Gd0.5MoO4: A novel highly-efficient infrared-to-visible upconversion material for optical temperature sensors and optical heaters. Chem. Eng. J. 306, 840–848. 10.1016/j.cej.2016.08.007

[B35] Du P. Yu J. S. (2017). Near-ultraviolet light induced visible emissions in Er3+-activated La2MoO6 nanoparticles for solid-state lighting and non-contact thermometry. Chem. Eng. J. 327, 109–119. 10.1016/j.cej.2017.06.069

[B36] Fang H. Li M. Liu Q. Gai Y. Yuan L. Wang S. (2020). Ultra-sensitive nanoprobe modified with tumor cell membrane for UCL/MRI/PET multimodality precise imaging of triple-negative breast cancer. Nano-Micro Lett. 12 (1), 62. 10.1007/s40820-020-0396-4 PMC7770711 34138297

[B37] Farah C. Michel L. Y. M. Balligand J.-L. (2018). Nitric oxide signalling in cardiovascular health and disease. Nat. Rev. Cardiol. 15 (5), 292–316. 10.1038/nrcardio.2017.224 29388567

[B38] Feng M. Lv R. Xiao L. Hu B. Zhu S. He F. (2018). Highly erbium-doped nanoplatform with enhanced red emission for dual-modal optical-imaging-guided photodynamic therapy. Inorg. Chem. 57 (23), 14594–14602. 10.1021/acs.inorgchem.8b02257 30444117

[B39] Gao G. Jiang Y.-W. Guo Y. Jia H.-R. Cheng X. Deng Y. (2020). Enzyme-mediated tumor starvation and phototherapy enhance mild-temperature photothermal therapy. Adv. Funct. Mater. 30 (16), 1909391. 10.1002/adfm.201909391

[B40] Gao X. Li T. He J. Ye K. Song X. Wang N. (2017). Synthesis of Yb3+, Ho3+ and Tm3+ co-doped beta-NaYF4 nanoparticles by sol-gel method and the multi-color upconversion luminescence properties. J. Mater. Science-Materials Electron. 28 (16), 11644–11653. 10.1007/s10854-017-6967-6

[B41] Gao Y. Li R. Zheng W. Shang X. Wei J. Zhang M. (2019). Broadband NIR photostimulated luminescence nanoprobes based on CaS:Eu2+, Sm3+ nanocrystals. Chem. Sci. 10 (21), 5452–5460. 10.1039/c9sc01321k 31293727 PMC6552487

[B42] Garfield D. J. Borys N. J. Hamed S. M. Torquato N. A. Tajon C. A. Tian B. (2018). Enrichment of molecular antenna triplets amplifies upconverting nanoparticle emission. Nat. Photonics 12 (7), 402–407. 10.1038/s41566-018-0156-x

[B43] Ge G. Lu Y. Qu X. Zhao W. Ren Y. Wang W. (2020). Muscle-inspired self-healing hydrogels for strain and temperature sensor. Acs Nano 14 (1), 218–228. 10.1021/acsnano.9b07874 31808670

[B44] Gerelkhuu Z. Huy B. T. Jung D. Sharipov M. Lee Y.-I. (2021). Selective optosensing of iron(III) ions in HeLa cells using NaYF4:Yb3+/Tm3+ upconversion nanoparticles coated with polyepinephrine. Anal. Bioanal. Chem. 413 (5), 1363–1371. 10.1007/s00216-020-03099-1 33388932

[B45] Gerelkhuu Z. Jung D. Bui The H. Tawfik S. M. Conte M. L. Conte E. D. (2019). Highly selective and sensitive detection of catecholamines using NaLuGdF4:Yb3+/Er3+ upconversion nanoparticles decorated with metal ions. Sensors Actuators B-Chemical 284, 172–178. 10.1016/j.snb.2018.12.135

[B46] Ghosh S. Chang Y.-F. Yang D.-M. Chattopadhyay S. (2020). Upconversion nanoparticle-mOrange protein FRET nanoprobes for self-ratiometric/ratiometric determination of intracellular pH, and single cell pH imaging. Biosens. Bioelectron. 155, 112115. 10.1016/j.bios.2020.112115 32217331

[B47] Gierschner J. Shi J. Milian-Medina B. Roca-Sanjuan D. Varghese S. Park S. (2021). Luminescence in crystalline organic materials: From molecules to molecular solids. Adv. Opt. Mater. 9 (13), 2002251. 10.1002/adom.202002251

[B48] Gong Z. Wu T. Chen X. Guo J. Zhang Y. Li Y. (2021). Upconversion nanoparticle decorated spider silks as single-cell thermometers. Nano Lett. 21 (3), 1469–1476. 10.1021/acs.nanolett.0c04644 33476159

[B49] Gu Y. Jiang Z. Ren D. Shang Y. Hu Y. Yi L. (2021). Electrochemiluminescence sensor based on the target recognition-induced aggregation of sensing units for Hg2+ determination. Sensors Actuators B-Chemical 337, 129821. 10.1016/j.snb.2021.129821

[B50] Guo S. Xie X. Huang L. Huang W. (2016). Sensitive water probing through nonlinear photon upconversion of lanthanide-doped nanoparticles. Acs Appl. Mater. Interfaces 8 (1), 847–853. 10.1021/acsami.5b10192 26651357

[B51] Guo T. Tang Q. Guo Y. Qiu H. Dai J. Xing C. (2021). Boron quantum dots for photoacoustic imaging-guided photothermal therapy. Acs Appl. Mater. Interfaces 13 (1), 306–311. 10.1021/acsami.0c21198 33382584

[B52] Guryev E. L. Shilyagina N. Y. Kostyuk A. B. Sencha L. M. Balalaeva I. V. Vodeneev V. A. (2019). Preclinical study of biofunctional polymer-coated upconversion nanoparticles. Toxicol. Sci. 170 (1), 123–132. 10.1093/toxsci/kfz086 30985900

[B53] Hamza M. F. Yap H. J. Choudhury I. A. (2017). Recent advances on the use of meta-heuristic optimization algorithms to optimize the type-2 fuzzy logic systems in intelligent control. Neural Comput. Appl. 28 (5), 979–999. 10.1007/s00521-015-2111-9

[B54] Han S. Deng R. Xie X. Liu X. (2014). Enhancing luminescence in lanthanide-doped upconversion nanoparticles. Angew. Chemie-International Ed. 53 (44), 11702–11715. 10.1002/anie.201403408 25204638

[B55] Hao T. Wu X. Xu L. Liu L. Ma W. Kuang H. (2017). Ultrasensitive detection of prostate-specific antigen and thrombin based on gold-upconversion nanoparticle assembled pyramids. Small 13 (19), 1603944. 10.1002/smll.201603944 28371262

[B56] Hassairi M. A. Garrido Hernandez A. Dammak M. Zambon D. Chadeyron G. Mahiou R. (2018). Tuning white upconversion emission in GdPO4:Er/Yb/Tm phosphors. J. Luminescence 203, 707–713. 10.1016/j.jlumin.2018.07.024

[B57] Hazra C. Ullah S. Serge Correales Y. E. Caetano L. G. Ribeiro S. J. L. (2018). Enhanced NIR-I emission from water-dispersible NIR-II dye-sensitized core/active shell upconverting nanoparticles. J. Mater. Chem. C 6 (17), 4777–4785. 10.1039/c8tc00335a

[B58] He Y. Guo S. Zhang Y. Liu Y. Ju H. (2021). Near-infrared photo-controlled permeability of a biomimetic polymersome with sustained drug release and efficient tumor therapy. Acs Appl. Mater. Interfaces 13 (13), 14951–14963. 10.1021/acsami.1c00842 33764734

[B59] Hopper E. Turton B. C. H. (2001). A review of the application of meta-heuristic algorithms to 2D strip packing problems. Artif. Intell. Rev. 16 (4), 257–300. 10.1023/a:1012590107280

[B60] Hou X. Tao Y. Pang Y. Li X. Jiang G. Liu Y. (2018). Nanoparticle-based photothermal and photodynamic immunotherapy for tumor treatment. Int. J. Cancer 143 (12), 3050–3060. 10.1002/ijc.31717 29981170

[B61] Hu C. He X. Chen Y. Yang X. Qin L. Lei T. (2021). Metformin mediated PD-L1 downregulation in combination with photodynamic-immunotherapy for treatment of breast cancer. Adv. Funct. Mater. 31 (11), 2007149. 10.1002/adfm.202007149

[B62] Hu Z. Fang C. Li B. Zhang Z. Cao C. Cai M. (2020). First-in-human liver-tumour surgery guided by multispectral fluorescence imaging in the visible and near-infrared-I/II windows. Nat. Biomed. Eng. 4 (3), 259–271. 10.1038/s41551-019-0494-0 31873212

[B63] Issa S. A. M. Sayyed M. I. Zaid M. H. M. Matori K. A. (2018). Photon parameters for gamma-rays sensing properties of some oxide of lanthanides. Results Phys. 9, 206–210. 10.1016/j.rinp.2018.02.039

[B64] Ji Y. Lu F. Hu W. Zhao H. Tang Y. Li B. (2019). Tandem activated photodynamic and chemotherapy: Using pH-Sensitive nanosystems to realize different tumour distributions of photosensitizer/prodrug for amplified combination therapy. Biomaterials 219, 119393. 10.1016/j.biomaterials.2019.119393 31382206

[B65] Jia T. Wang Z. Sun Q. Dong S. Xu J. Zhang F. (2020). Intelligent Fe-Mn layered double hydroxides nanosheets anchored with upconversion nanoparticles for oxygen-elevated synergetic therapy and bioimaging. Small 16 (46), 2001343. 10.1002/smll.202001343 33107221

[B66] Kamata-Sakurai M. Narita Y. Hori Y. Nemoto T. Uchikawa R. Honda M. (2021). Antibody to CD137 activated by extracellular adenosine triphosphate is tumor selective and broadly effective *in vivo* without systemic immune activation. Cancer Discov. 11 (1), 158–175. 10.1158/2159-8290.cd-20-0328 32847940

[B67] Katoch S. Chauhan S. S. Kumar V. (2021). A review on genetic algorithm: Past, present, and future. Multimedia Tools Appl. 80 (5), 8091–8126. 10.1007/s11042-020-10139-6 PMC7599983 33162782

[B68] Kobayashi H. Furusawa A. Rosenberg A. Choyke P. L. (2021). Near-infrared photoimmunotherapy of cancer: A new approach that kills cancer cells and enhances anti-cancer host immunity. Int. Immunol. 33 (1), 7–15. 10.1093/intimm/dxaa037 32496557 PMC7771006

[B69] Kovalenko M. V. Scheele M. Talapin D. V. (2009). Colloidal nanocrystals with molecular metal chalcogenide surface ligands. Science 324 (5933), 1417–1420. 10.1126/science.1170524 19520953

[B70] Kovalenko M. V. Spokoyny B. Lee J. S. Scheele M. Weber A. Perera S. (2010). Semiconductor nanocrystals functionalized with antimony telluride zintl ions for nanostructured thermoelectrics. J. Am. Chem. Soc. 132 (19), 6686–6695. 10.1021/ja909591x 20423085

[B71] Kumar R. Binetti L. Nguyen T. H. Alwis L. S. M. Sun T. Grattan K. T. V. (2020). Optical fibre thermometry using ratiometric green emission of an upconverting nanoparticle-polydimethylsiloxane composite. Sensors Actuators a-Physical 312, 112083. 10.1016/j.sna.2020.112083

[B72] Lan G. Ni K. Lin W. (2019). Nanoscale metal-organic frameworks for phototherapy of cancer. Coord. Chem. Rev. 379, 65–81. 10.1016/j.ccr.2017.09.007 30739946 PMC6366651

[B73] Laurenti M. Paez-Perez M. Algarra M. Alonso-Cristobal P. Lopez-Cabarcos E. Mendez-Gonzalez D. (2016). Enhancement of the upconversion emission by visible-to-near-infrared fluorescent graphene quantum dots for miRNA detection. Acs Appl. Mater. Interfaces 8 (20), 12644–12651. 10.1021/acsami.6b02361 27153453 PMC5058637

[B74] Lee H. R. Kim D. W. Jones V. O. Choi Y. Ferry V. E. Geller M. A. (2021). Sonosensitizer-functionalized graphene nanoribbons for adhesion blocking and sonodynamic ablation of ovarian cancer spheroids. Adv. Healthc. Mater. 10, 2001368. 10.1002/adhm.202001368 PMC8550295 34050609

[B75] Li C. Lin J. (2010). Rare Earth fluoride nano-/microcrystals: Synthesis, surface modification and application. J. Mater. Chem. 20 (33), 6831–6847. 10.1039/c0jm00031k

[B76] Li F. Li C. Liu X. Chen Y. Bai T. Wang L. (2012). Hydrophilic, upconverting, multicolor, lanthanide-doped NaGdF4 nanocrystals as potential multifunctional bioprobes. Chemistry-a Eur. J. 18 (37), 11641–11646. 10.1002/chem.201201309 22886785

[B77] Li G. Tian Y. Zhao Y. Lin J. (2015). Recent progress in luminescence tuning of Ce3+ and Eu2+-activated phosphors for pc-WLEDs. Chem. Soc. Rev. 44 (23), 8688–8713. 10.1039/c4cs00446a 26421319

[B78] Li H. Tan M. Wang X. Li F. Zhang Y. Zhao L. (2020). Temporal multiplexed *in vivo* upconversion imaging. J. Am. Chem. Soc. 142 (4), 2023–2030. 10.1021/jacs.9b11641 31910008

[B79] Li H. Wang C. Jiang Y. Cui Z. Lin Q. (2014). One-step synthesis of biocompatible chitosan/nagdf4:Eu3+ nanocomposite with fluorescent and magnetic properties for bioimaging. Nano 9 (1), 1450007. 10.1142/s1793292014500076

[B80] Li J. Rao J. Pu K. (2018). Recent progress on semiconducting polymer nanoparticles for molecular imaging and cancer phototherapy. Biomaterials 155, 217–235. 10.1016/j.biomaterials.2017.11.025 29190479 PMC5978728

[B81] Li N. Tan H. Xie S. Liu S. Tong C. Ouyang M. (2020). Hexadecylpyridinium chloride mediated hydrothermal synthesis of NaYF4:Yb,Er nanocrystal and their luminescent properties. J. Nanosci. Nanotechnol. 20 (3), 1866–1872. 10.1166/jnn.2020.17348 31492354

[B82] Li Q. Wang Z. Chen Y. Zhang G. (2017). Elemental bio-imaging of PEGylated NaYF4:Yb/Tm/Gd upconversion nanoparticles in mice by laser ablation inductively coupled plasma mass spectrometry to study toxic side effects on the spleen, liver and kidneys. Metallomics 9 (8), 1150–1156. 10.1039/c7mt00132k 28745365

[B83] Li S. Xu L. Ma W. Wu X. Sun M. Kuang H. (2016). Dual-mode ultrasensitive quantification of MicroRNA in living cells by chiroplasmonic nanopyramids self-assembled from gold and upconversion nanoparticles. J. Am. Chem. Soc. 138 (1), 306–312. 10.1021/jacs.5b10309 26691742

[B84] Li X. Zhang F. Zhao D. (2015). Lab on upconversion nanoparticles: Optical properties and applications engineering via designed nanostructure. Chem. Soc. Rev. 44 (6), 1346–1378. 10.1039/c4cs00163j 25052250

[B85] Li Y. Li Y. Wang R. Xu Y. Zheng W. (2017). Enhancing upconversion luminescence by annealing processes and the high-temperature sensing of ZnO:Yb/Tm nanoparticles. New J. Chem. 41 (15), 7116–7122. 10.1039/c7nj01358b

[B86] Li Y. Liu J. Qin X. Deng Y. Zhang J. Sun Y. (2019). Ultrafast synthesis of fluorine-18 doped bismuth based upconversion nanophosphors for tri-modal CT/PET/UCL imaging *in vivo* . Chem. Commun. 55 (50), 7259–7262. 10.1039/c9cc02677k 31168526

[B87] Li Z.-H. Chen Y. Sun Y. Zhang X.-Z. (2021). Platinum-doped prussian blue nanozymes for multiwavelength bioimaging guided photothermal therapy of tumor and anti-inflammation. Acs Nano 15 (3), 5189–5200. 10.1021/acsnano.0c10388 33703878

[B88] Li Z. Zhang Y. (2006). Monodisperse silica-coated polyvinylpyrrolidone/NaYF(4) nanocrystals with multicolor upconversion fluorescence emission. Angew. Chem. Int. Ed. Engl. 45 (46), 7732–7735. 10.1002/anie.200602975 17089426

[B89] Liang T. Wang Q. Li Z. Wang P. Wu J. Zuo M. (2020). Removing the obstacle of dye-sensitized upconversion luminescence in aqueous phase to achieve high-contrast deep imaging *in vivo* . Adv. Funct. Mater. 30 (16), 1910765. 10.1002/adfm.201910765

[B90] Liang X. Fan J. Zhao Y. Jin R. (2020). Core-shell structured NaYF4:Yb,Er nanoparticles with excellent upconversion luminescent for targeted drug delivery. J. Clust. Sci. 32, 1683–1691. 10.1007/s10876-020-01929-x

[B91] Lin B. Liu J. Wang Y. Yang F. Huang L. Lv R. (2020). Enhanced upconversion luminescence-guided synergistic antitumor therapy based on photodynamic therapy and immune checkpoint blockade. Chem. Mater. 32 (11), 4627–4640. 10.1021/acs.chemmater.0c01031

[B92] Lin M. Xie L. Wang Z. Richards B. S. Gao G. Zhong J. (2019). Facile synthesis of mono-disperse sub-20 nm NaY(WO4)(2):Er3+,Yb3+ upconversion nanoparticles: A new choice for nanothermometry. J. Mater. Chem. C 7 (10), 2971–2977. 10.1039/c8tc05669b

[B93] Lin M. Zhao Y. Wang S. Liu M. Duan Z. Chen Y. (2012). Recent advances in synthesis and surface modification of lanthanide-doped upconversion nanoparticles for biomedical applications. Biotechnol. Adv. 30 (6), 1551–1561. 10.1016/j.biotechadv.2012.04.009 22561011

[B94] Lin M. Zou S. Liao X. Chen Y. Luo D. Ji L. (2021). Ruthenium(ii) complexes as bioorthogonal two-photon photosensitizers for tumour-specific photodynamic therapy against triple-negative breast cancer cells. Chem. Commun. 57 (36), 4408–4411. 10.1039/d1cc00661d 33949487

[B95] Liu B. Sun J. Zhu J. Li B. Ma C. Gu X. (2020). Injectable and NIR-responsive DNA-inorganic hybrid hydrogels with outstanding photothermal therapy. Adv. Mater. 32 (39), 2004460. 10.1002/adma.202004460 32830376

[B96] Liu J. Yang F. Feng M. Wang Y. Peng X. Lv R. (2019). Surface plasmonic enhanced imaging-guided photothermal/photodynamic therapy based on lanthanide-metal nanocomposites under single 808 nm laser. Acs Biomaterials Sci. Eng. 5 (10), 5051–5059. 10.1021/acsbiomaterials.9b01112 33455252

[B97] Liu K. Liu X. Zeng Q. Zhang Y. Tu L. Liu T. (2012). Covalently assembled NIR nanoplatform for simultaneous fluorescence imaging and photodynamic therapy of cancer cells. Acs Nano 6 (5), 4054–4062. 10.1021/nn300436b 22463487

[B98] Liu L. Li X. T. Zhang H. Chen H. D. Abualrejal M. M. A. Song D. Q. (2021). Six-in-one peptide functionalized upconversion@polydopamine nanoparticle-based ratiometric fluorescence sensing platform for real-time evaluating anticancer efficacy through monitoring caspase-3 activity. Sensors Actuators B-Chemical 333, 129554. 10.1016/j.snb.2021.129554

[B99] Liu Q. Xu M. Yang T. Tian B. Zhang X. Li F. (2018). Highly photostable near-IR-excitation upconversion nanocapsules based on triplet-triplet annihilation for *in vivo* bioimaging application. Acs Appl. Mater. Interfaces 10 (12), 9883–9888. 10.1021/acsami.7b17929 29425018

[B100] Liu S. Liu S. Ming H. Du F. Peng J. You W. (2018). Tunable multicolor and bright white upconversion luminescence in Er3+/Tm3+/Yb3+ tri-doped SrLu2O4 phosphors. J. Mater. Sci. 53 (20), 14469–14484. 10.1007/s10853-018-2632-6

[B101] Liu Y. Chen M. Zhao Y. Lv S. Zheng D. Liu D. (2021). A novel D-A-D photosensitizer for efficient NIR imaging and photodynamic therapy. Chembiochem 22, 2161–2167. 10.1002/cbic.202100107 33871143

[B102] Liu Y. Meng X. Bu W. (2019). Upconversion-based photodynamic cancer therapy. Coord. Chem. Rev. 379, 82–98. 10.1016/j.ccr.2017.09.006

[B103] Lu C. Guan J. Lu S. Jin Q. Rousseau B. Lu T. (2021). DNA sensing in mismatch repair-deficient tumor cells is essential for anti-tumor immunity. Cancer Cell 39 (1), 96–108.e6. 10.1016/j.ccell.2020.11.006 33338425 PMC9477183

[B104] Lundberg J. O. Gladwin M. T. Weitzberg E. (2015). Strategies to increase nitric oxide signalling in cardiovascular disease. Nat. Rev. Drug Discov. 14 (9), 623–641. 10.1038/nrd4623 26265312

[B105] Luo R. Chen L. Li Q. Zhou J. Mei L. Ning Z. (2020). Bi3+-Doped BaYF5:Yb,Er upconversion nanoparticles with enhanced luminescence and application case for X-ray computed tomography imaging. Inorg. Chem. 59 (24), 17906–17915. 10.1021/acs.inorgchem.0c01818 33252238

[B106] Lutken C. D. Achiam M. P. Osterkamp J. Svendsen M. B. Nerup N. (2021). Quantification of fluorescence angiography: Toward a reliable intraoperative assessment of tissue perfusion-A narrative review. Langenbecks Archives Surg. 406 (2), 251–259. 10.1007/s00423-020-01966-0 32821959

[B107] Lv R. Feng M. Parak W. J. (2018). Up-conversion luminescence properties of lanthanide-gold hybrid nanoparticles as analyzed with Discrete Dipole approximation. Nanomaterials 8 (12), 989. 10.3390/nano8120989 30501026 PMC6315549

[B108] Lv R. Xiao L. Jiang X. Feng M. Yang F. Tian J. (2018). Optimization of red luminescent intensity in Eu3+-doped lanthanide phosphors using genetic algorithm. Acs Biomaterials Sci. Eng. 4 (12), 4378–4384. 10.1021/acsbiomaterials.8b00513 33418830

[B109] Lv R. Xiao L. Wang Y. Yang F. Tian J. Lin J. (2019). Searching for the optimized luminescent lanthanide phosphor using heuristic algorithms. Inorg. Chem. 58 (9), 6458–6466. 10.1021/acs.inorgchem.9b00667 31016972

[B110] Lv R. Yang F. Jiang X. Hu B. Zhang X. Chen X. (2020). Plasmonic modulated upconversion fluorescence by adjustable distributed gold nanoparticles. J. Luminescence 220, 116974. 10.1016/j.jlumin.2019.116974

[B111] Lv R. Yang G. Dai Y. Gai S. He F. Yang P. (2014). Self-produced bubble-template synthesis of La2O3:Yb/Er@Au hollow spheres with markedly enhanced luminescence and release properties. Crystengcomm 16 (41), 9612–9621. 10.1039/c4ce01063a

[B112] Lv R. Yang P. Dai Y. Gai S. He F. Lin J. (2014). Lutecium fluoride hollow mesoporous spheres with enhanced up-conversion luminescent bioimaging and light-triggered drug release by gold nanocrystals. ACS Appl. Mater. interfaces 6 (17), 15550–15563. 10.1021/am504347e 25138031

[B113] Ma L. Liu F. Lei Z. Wang Z. (2017). A novel upconversion@polydopamine core@shell nanoparticle based aptameric biosensor for biosensing and imaging of cytochrome c inside living cells. Biosens. Bioelectron. 87, 638–645. 10.1016/j.bios.2016.09.017 27619527

[B114] Ma S. Wang L. Liu Z. Luo X. Zhou Z. Xie J. (2021). One stone, two birds": Engineering 2-D ultrathin heterostructure nanosheet BiNS@NaLnF(4) for dual-modal computed tomography/magnetic resonance imaging guided, photonic synergetic theranostics. Nanoscale 13 (1), 185–194. 10.1039/d0nr07590f 33325961

[B115] Ma Y. Ma Y. Gao M. Han Z. Jiang W. Gu Y. (2021). Platelet-mimicking therapeutic system for noninvasive mitigation of the progression of atherosclerotic plaques. Adv. Sci. 8 (8), 2004128. 10.1002/advs.202004128 PMC8061396 33898191

[B116] Mahata M. K. Bae H. Lee K. T. (2017). Upconversion luminescence sensitized pH-nanoprobes. Molecules 22 (12), 2064. 10.3390/molecules22122064 29186844 PMC6149687

[B117] Maret W. (2013). Zinc biochemistry: From a single zinc enzyme to a Key element of life. Adv. Nutr. 4 (1), 82–91. 10.3945/an.112.003038 23319127 PMC3648744

[B118] Mates J. M. Segura J. A. Alonso F. J. Marquez J. (2012). Oxidative stress in apoptosis and cancer: An update. Archives Toxicol. 86 (11), 1649–1665. 10.1007/s00204-012-0906-3 22811024

[B119] Mintz K. J. Zhou Y. Leblanc R. M. (2019). Recent development of carbon quantum dots regarding their optical properties, photoluminescence mechanism, and core structure. Nanoscale 11 (11), 4634–4652. 10.1039/c8nr10059d 30834912 PMC6467229

[B120] Mir W. J. Sheikh T. Arfin H. Xia Z. Nag A. (2020). Lanthanide doping in metal halide perovskite nanocrystals: Spectral shifting, quantum cutting and optoelectronic applications. Npg Asia Mater. 12 (1), 9. 10.1038/s41427-019-0192-0

[B121] Mousavand T. Ohara S. Naka T. Umetsu M. Takami S. Adschiri T. (2010). Organic-ligand-assisted hydrothermal synthesis of ultrafine and hydrophobic ZnO nanoparticles. J. Mater. Res. 25 (2), 219–223. 10.1557/jmr.2010.0037

[B122] Mpekris F. Voutouri C. Baish J. W. Duda D. G. Munn L. L. Stylianopoulos T. (2020). Combining microenvironment normalization strategies to improve cancer immunotherapy. Proc. Natl. Acad. Sci. U. S. A. 117 (7), 3728–3737. 10.1073/pnas.1919764117 32015113 PMC7035612

[B123] Mykhaylyk O. Cherchenko A. Ilkin A. Dudchenko N. Ruditsa V. Novoseletz M. (2001). Glial brain tumor targeting of magnetite nanoparticles in rats. J. MAGNETISM MAGNETIC Mater. 225 (1-2), 241–247. 10.1016/s0304-8853(00)01264-6

[B124] Ozdemir G. Karaboga N. (2019). A review on the cosine modulated filter bank studies using meta-heuristic optimization algorithms. Artif. Intell. Rev. 52 (3), 1629–1653. 10.1007/s10462-017-9595-x

[B125] Pan G. Bai X. Yang D. Chen X. Jing P. Qu S. (2017). Doping lanthanide into perovskite nanocrystals: Highly improved and expanded optical properties. Nano Lett. 17 (12), 8005–8011. 10.1021/acs.nanolett.7b04575 29182877

[B126] Pang H. Tian C. He G. Zhang D. Yang J. Zhang Q. (2021). NIR-absorbing Prussian blue nanoparticles for transarterial infusion photothermal therapy of VX2 tumors implanted in rabbits. Nanoscale 13 (18), 8490–8497. 10.1039/d1nr01394g 33913450

[B127] Park J. Xu M. Li F. Zhou H.-C. (2018). 3D long-range triplet migration in a water-stable metal-organic framework for upconversion-based ultralow-power *in vivo* imaging. J. Am. Chem. Soc. 140 (16), 5493–5499. 10.1021/jacs.8b01613 29634258

[B128] Pedziwiatr-Werbicka E. Horodecka K. Shcharbin D. Bryszewska M. (2021). Nanoparticles in combating cancer: Opportunities and limitations: A brief review. Curr. Med. Chem. 28 (2), 346–359. 10.2174/1875533xmta0kmdkhw 32000637

[B129] Peng J. Xu W. Teoh C. L. Han S. Kim B. Samanta A. (2015). High-efficiency *in vitro* and *in vivo* detection of Zn2+ by dye-assembled upconversion nanoparticles. J. Am. Chem. Soc. 137 (6), 2336–2342. 10.1021/ja5115248 25626163

[B130] Pfeiffer F. (2018). X-ray ptychography. Nat. Photonics 12 (1), 9–17. 10.1038/s41566-017-0072-5

[B131] Pinto A. Marangon I. Mereaux J. Nicolas-Boluda A. Lavieu G. Wilhelm C. (2021). Immune reprogramming precision photodynamic therapy of peritoneal metastasis by scalable stem-cell-derived extracellular vesicles. Acs Nano 15 (2), 3251–3263. 10.1021/acsnano.0c09938 33481565

[B132] Plattner N. Noe F. (2015). Protein conformational plasticity and complex ligand-binding kinetics explored by atomistic simulations and Markov models. Nat. Commun. 6, 7653. 10.1038/ncomms8653 26134632 PMC4506540

[B133] Qiao Y. F. Qiao S. Q. Yu X. Min Q. H. Pi C. J. Qiu J. B. (2021). Plant tissue imaging with bipyramidal upconversion nanocrystals by introducing Tm3+ ions as energy trapping centers. Nanoscale 13 (17), 8181–8187. 10.1039/d0nr07399g 33884383

[B134] Qiu X. Zhu X. Xu M. Yuan W. Feng W. Li F. (2017). Hybrid nanoclusters for near-infrared to near-infrared upconverted persistent luminescence bioimaging. Acs Appl. Mater. Interfaces 9 (38), 32583–32590. 10.1021/acsami.7b10618 28856891

[B135] Qu A. Wu X. Xu L. Liu L. Ma W. Kuang H. (2017). SERS- and luminescence-active Au-Au-UCNP trimers for attomolar detection of two cancer biomarkers. Nanoscale 9 (11), 3865–3872. 10.1039/c6nr09114h 28252127

[B136] Quan L. N. de Arquer F. P. G. Sabatini R. P. Sargent E. H. (2018). Perovskites for light emission. Adv. Mater. 30 (45), 1801996. 10.1002/adma.201801996 30160805

[B137] Radunz S. Andresen E. Wuerth C. Koerdt A. Tschiche H. R. Resch-Genger U. (2019). Simple self-referenced luminescent pH sensors based on upconversion nanocrystals and pH-sensitive fluorescent BODIPY dyes. Anal. Chem. 91 (12), 7756–7764. 10.1021/acs.analchem.9b01174 31091879

[B138] Rafique R. Baek S. H. Le Minh Tu P. Chang S.-J. Gul A. R. Park T. J. (2019). A facile hydrothermal synthesis of highly luminescent NaYF4:Yb3+/Er3+ upconversion nanoparticles and their biomonitoring capability. Mater. Sci. Eng. C-Materials Biol. Appl. 99, 1067–1074. 10.1016/j.msec.2019.02.046 30889639

[B139] Rafique R. Baek S. H. Park C. Y. Chang S. J. Gul A. R. Ha S. (2018). Morphological evolution of upconversion nanoparticles and their biomedical signal generation. Sci. Rep. 8 (1), 17101. 10.1038/s41598-018-35513-1 30459423 PMC6244231

[B140] Ravichandran V. Thuy Giang Nguyen C. Dae Gun C. Han Chang K. Min Suk S. (2020). Non-ionic polysorbate-based nanoparticles for efficient combination chemo/photothermal/photodynamic therapy. J. Industrial Eng. Chem. 88, 260–267. 10.1016/j.jiec.2020.04.023

[B141] Reddy K. L. Balaji R. Kumar A. Krishnan V. (2018). Lanthanide doped near infrared active upconversion nanophosphors: Fundamental concepts, synthesis strategies, and technological applications. Small 14 (37), 1801304. 10.1002/smll.201801304 30066489

[B142] Sangkhae V. Nemeth E. (2017). Regulation of the iron homeostatic hormone hepcidin. Adv. Nutr. 8 (1), 126–136. 10.3945/an.116.013961 28096133 PMC5227985

[B143] Shahzad M. K. Zhang Y. Raza A. Ikram M. Qi K. Khan M. U. (2019). Polymer microfibers incorporated with silver nanoparticles: A new platform for optical sensing. Nanoscale Res. Lett. 14 (1), 270. 10.1186/s11671-019-3108-6 31396725 PMC6687803

[B144] Shan X. Chen Q. Yin X. Jiang C. Li T. Wei S. (2020). Polypyrrole-based double rare Earth hybrid nanoparticles for multimodal imaging and photothermal therapy. J. Mater Chem. B 8 (3), 426–437. 10.1039/c9tb02254f 31833528

[B145] Shanmugam V. Selvakumar S. Yeh C.-S. (2014). Near-infrared light-responsive nanomaterials in cancer therapeutics. Chem. Soc. Rev. 43 (17), 6254–6287. 10.1039/c4cs00011k 24811160

[B146] Shen Y. Wu T. Wang Y. Zhang S.-L. Zhao X. Chen H.-Y. (2021). Nucleolin-targeted ratiometric fluorescent carbon dots with a remarkably large emission wavelength shift for precise imaging of cathepsin B in living cancer cells. Anal. Chem. 93 (8), 4042–4050. 10.1021/acs.analchem.0c05046 33586959

[B147] Shi M. Fu Z. Pan W. Chen Y. Wang K. Zhou P. (2021). A protein-binding molecular photothermal agent for tumor ablation. Angew. Chemie-International Ed. 60 (24), 13676–13680. 10.1002/ange.202101009 33783939

[B148] Su J. Li Y. Gu W. Liu X. (2020). Spiropyran-modified upconversion nanocomposite as a fluorescent sensor for diagnosis of histidinemia. Rsc Adv. 10 (45), 26664–26670. 10.1039/d0ra03711g 35515791 PMC9055446

[B149] Suman B. Kumar P. (2006). A survey of simulated annealing as a tool for single and multiobjective optimization. J. Operational Res. Soc. 57 (10), 1143–1160. 10.1057/palgrave.jors.2602068

[B150] Sun J.-S. Li S.-W. Shi L.-L. Zhou T.-M. Li X.-P. Zhang J.-S. (2015). Experimental optimal design of the Er3+/Yb3+ codoped BaGd2ZnO5 phosphor and its upconversion luminescence properties. Acta Phys. Sin. 64 (24), 243301. 10.7498/aps.64.243301

[B151] Sun Y. Peng J. Feng W. Li F. (2013). Upconversion nanophosphors naluf(4): Yb, Tm for lymphatic imaging *in vivo* by real-time upconversion luminescence imaging under ambient light and high-resolution X-ray CT. Theranostics 3 (5), 346–353. 10.7150/thno.5137 23650481 PMC3645060

[B152] Sun Y. Yu M. X. Liang S. Zhang Y. J. Li C. G. Mou T. T. (2011). Fluorine-18 labeled rare-Earth nanoparticles for positron emission tomography (PET) imaging of sentinel lymph node. Biomaterials 32 (11), 2999–3007. 10.1016/j.biomaterials.2011.01.011 21295345

[B153] Sun Y. Zhang Y. Gao Y. Wang P. He G. Blum N. T. (2020). Six birds with one stone: Versatile nanoporphyrin for single-laser-triggered synergistic phototheranostics and robust immune activation. Adv. Mater. 32 (48), 2004481. 10.1002/adma.202004481 33015905

[B154] Tan M. Li F. Cao N. Li H. Wang X. Zhang C. (2020). Accurate *in vivo* nanothermometry through NIR-II lanthanide luminescence lifetime. Small 16 (48), 2004118. 10.1002/smll.202004118 33155363

[B155] Tan S. Li D. Zhu X. (2020). Cancer immunotherapy: Pros, cons and beyond. Biomed. Pharmacother. 124, 109821. 10.1016/j.biopha.2020.109821 31962285

[B156] Tangirala R. Baker J. L. Alivisatos A. P. Milliron D. J. (2010). Modular inorganic nanocomposites by conversion of nanocrystal superlattices. Angew. Chem. Int. Ed. Engl. 49 (16), 2878–2882. 10.1002/anie.200906642 20301153

[B157] Tay R. E. Richardson E. K. Toh H. C. (2021). Revisiting the role of CD4(+)T cells in cancer immunotherapy-new insights into old paradigms. Cancer Gene Ther. 28 (1-2), 5–17. 10.1038/s41417-020-0183-x 32457487 PMC7886651

[B158] Teng C. W. Huang V. Arguelles G. R. Zhou C. Cho S. S. Harmsen S. (2021). Applications of indocyanine green in brain tumor surgery: Review of clinical evidence and emerging technologies. Neurosurg. Focus 50 (1), E4. 10.3171/2020.10.focus20782 33386005

[B159] Thangaraj R. Pant M. Abraham A. Bouvry P. (2011). Particle swarm optimization: Hybridization perspectives and experimental illustrations. Appl. Math. Comput. 217 (12), 5208–5226. 10.1016/j.amc.2010.12.053

[B160] Thariani R. Yager P. (2010). Imaging of surfaces by concurrent surface plasmon resonance and surface plasmon resonance-enhanced fluorescence. Plos One 5 (3), e9833. 10.1371/journal.pone.0009833 20360841 PMC2845608

[B161] Tian J. Huang B. Nawaz M. H. Zhang W. (2020). Recent advances of multi-dimensional porphyrin-based functional materials in photodynamic therapy. Coord. Chem. Rev. 420, 213410. 10.1016/j.ccr.2020.213410

[B162] Tian R. Zhao S. Liu G. Chen H. Ma L. You H. (2019). Construction of lanthanide-doped upconversion nanoparticle-Uelx Europaeus Agglutinin-I bioconjugates with brightness red emission for ultrasensitive *in vivo* imaging of colorectal tumor. Biomaterials 212, 64–72. 10.1016/j.biomaterials.2019.05.010 31103947

[B163] Tirotta I. Dichiarante V. Pigliacelli C. Cavallo G. Terraneo G. Bombelli F. B. (2015). ^19^F magnetic resonance imaging (MRI): From design of materials to clinical applications. Chem. Rev. 115 (2), 1106–1129. 10.1021/cr500286d 25329814

[B164] Tran Quang T. Ramasundaram S. Hwang B.-U. Lee N.-E. (2016). An all-elastomeric transparent and stretchable temperature sensor for body-attachable wearable electronics. Adv. Mater. 28 (3), 502–509. 10.1002/adma.201504441 26607674

[B165] Tsang M.-K. Bai G. Hao J. (2015). Stimuli responsive upconversion luminescence nanomaterials and films for various applications. Chem. Soc. Rev. 44 (6), 1585–1607. 10.1039/c4cs00171k 25200182

[B166] Tse W. H. Chen L. McCurdy C. M. Tarapacki C. M. Chronik B. A. Zhang J. (2019). Development of biocompatible NaGdF4: Er3+, Yb3+ upconversion nanoparticles used as contrast agents for bio-imaging. Can. J. Chem. Eng. 97 (10), 2678–2684. 10.1002/cjce.23510

[B167] Vedunova M. V. Mishchenko T. A. Mitroshina E. V. Ponomareva N. V. Yudintsev A. V. Generalova A. N. (2016). Cytotoxic effects of upconversion nanoparticles in primary hippocampal cultures. RSC Adv. 6 (40), 33656–33665. 10.1039/c6ra01272h

[B168] Wallis C. J. D. Butaney M. Satkunasivam R. Freedland S. J. Patel S. P. Hamid O. (2019). Association of patient sex with efficacy of immune checkpoint inhibitors and overall survival in advanced cancers A systematic review and meta-analysis. Jama Oncol. 5 (4), 529–536. 10.1001/jamaoncol.2018.5904 30605213 PMC6459215

[B169] Wang C. Zhao P. Yang G. Chen X. Jiang Y. Jiang X. (2020). Reconstructing the intracellular pH microenvironment for enhancing photodynamic therapy. Mater. Horizons 7 (4), 1180–1185. 10.1039/c9mh01824g

[B170] Wang F. Deng R. Liu X. (2014). Preparation of core-shell NaGdF4 nanoparticles doped with luminescent lanthanide ions to be used as upconversion-based probes. Nat. Protoc. 9 (7), 1634–1644. 10.1038/nprot.2014.111 24922272

[B171] Wang G. Peng Q. Li Y. (2011). Lanthanide-doped nanocrystals: Synthesis, optical-magnetic properties, and applications. Accounts Chem. Res. 44 (5), 322–332. 10.1021/ar100129p 21395256

[B172] Wang M. Song J. Zhou F. Hoover A. R. Murray C. Zhou B. (2019). NIR-triggered phototherapy and immunotherapy via an antigen-capturing nanoplatform for metastatic cancer treatment. Adv. Sci. 6 (10), 1802157. 10.1002/advs.201802157 PMC6523374 31131193

[B173] Wang X. Chang H. Xie J. Zhao B. Liu B. Xu S. (2014). Recent developments in lanthanide-based luminescent probes. Coord. Chem. Rev. 273, 201–212. 10.1016/j.ccr.2014.02.001

[B174] Wang X. Liu Q. Bu Y. Liu C.-S. Liu T. Yan X. (2015). Optical temperature sensing of rare-Earth ion doped phosphors. Rsc Adv. 5 (105), 86219–86236. 10.1039/c5ra16986k

[B175] Wang X. Zhuang J. Peng Q. Li Y. D. (2005). A general strategy for nanocrystal synthesis. Nature 437 (7055), 121–124. 10.1038/nature03968 16136139

[B176] Wang Z. Liu C. Zhao Y. Hu M. Ma D. Zhang P. (2019). Photomagnetic nanoparticles in dual-modality imaging and photo-sonodynamic activity against bacteria. Chem. Eng. J. 356, 811–818. 10.1016/j.cej.2018.09.077

[B177] Wattanasiritham L. Theerakulkait C. Wickramasekara S. Maier C. S. Stevens J. F. (2016). Isolation and identification of antioxidant peptides from enzymatically hydrolyzed rice bran protein. Food Chem. 192, 156–162. 10.1016/j.foodchem.2015.06.057 26304333

[B178] Wei R. Dong Y. Tu Y. Luo S. Pang X. Zhang W. (2021). Bioorthogonal pretargeting strategy for anchoring activatable photosensitizers on plasma membranes for effective photodynamic therapy. Acs Appl. Mater. Interfaces 13 (12), 14004–14014. 10.1021/acsami.1c01259 33728894

[B179] White K. A. Grillo-Hill B. K. Barber D. L. (2017). Cancer cell behaviors mediated by dysregulated pH dynamics at a glance. J. Cell Sci. 130 (4), 663–669. 10.1242/jcs.195297 28202602 PMC5339414

[B180] Wong Y.-T. Pang S.-Y. Tsang M.-K. Liu Y. Huang H. Yu S.-F. (2019). Electrochemically assisted flexible lanthanide upconversion luminescence sensing of heavy metal contamination with high sensitivity and selectivity. Nanoscale Adv. 1 (1), 265–272. 10.1039/c8na00012c 36132455 PMC9473281

[B181] Wu C. Huang X. Tang Y. Xiao W. Sun L. Shao J. (2019). Pyrrolopyrrole aza-BODIPY near-infrared photosensitizer for dual-mode imaging-guided photothermal cancer therapy. Chem. Commun. 55 (6), 790–793. 10.1039/c8cc07768a 30569923

[B182] Wu M. Liu X. Chen H. Duan Y. Liu J. Pan Y. (2021). Activation of pyroptosis by membrane-anchoring AIE photosensitizer design: New prospect for photodynamic cancer cell ablation. Angew. Chemie-International Ed. 60 (16), 9175–9180. 10.1002/ange.202016399 33543534

[B183] Wu M. Wang X. Wang K. Guo Z. (2017). An ultrasensitive fluorescent nanosensor for trypsin based on upconversion nanoparticles. Talanta 174, 797–802. 10.1016/j.talanta.2017.07.013 28738656

[B184] Wu X. Lee H. Bilsel O. Zhang Y. Li Z. Chen T. (2015). Tailoring dye-sensitized upconversion nanoparticle excitation bands towards excitation wavelength selective imaging. Nanoscale 7 (44), 18424–18428. 10.1039/c5nr05437k 26499208 PMC4636449

[B185] Wu X. Zhang Y. Takle K. Bilsel O. Li Z. Lee H. (2016). Dye-sensitized core/active shell upconversion nanoparticles for optogenetics and bioimaging applications. Acs Nano 10 (1), 1060–1066. 10.1021/acsnano.5b06383 26736013 PMC4913696

[B186] Xiao Q. Li Y. Li F. Zhang M. Zhang Z. Lin H. (2014). Rational design of a thermalresponsive-polymer-switchable FRET system for enhancing the temperature sensitivity of upconversion nanophosphors. Nanoscale 6 (17), 10179–10186. 10.1039/c4nr02497d 25046250

[B187] Xie Z. Fan T. An J. Choi W. Duo Y. Ge Y. (2020). Emerging combination strategies with phototherapy in cancer nanomedicine. Chem. Soc. Rev. 49 (22), 8065–8087. 10.1039/d0cs00215a 32567633

[B188] Xu D. Liu J. Wang Y. Jian Y. Wu W. Lv R. (2020). Black phosphorus nanosheet with high thermal conversion efficiency for photodynamic/photothermal/immunotherapy. Acs Biomaterials Sci. Eng. 6 (9), 4940–4948. 10.1021/acsbiomaterials.0c00984 33455288

[B189] Xu J. Lv R. Du S. Gai S. He F. Yang D. (2016). UCNPs@gelatin-ZnPc nanocomposite: Synthesis, imaging and anticancer properties. J. Mater Chem. B 4 (23), 4138–4146. 10.1039/c6tb00714g 32264616

[B190] Xu X. Sun Y. Zhang Q. Cai H. Li Q. Zhou S. (2019). Synthesis and photocatalytic activity of plasmon-enhanced core-shell upconversion luminescent photocatalytic Ag@SiO2@YF3:Ho3+@TiO2 nanocomposites. Opt. Mater. 94, 444–453. 10.1016/j.optmat.2019.05.038

[B191] Yakavets I. Francois A. Lamy L. Piffoux M. Gazeau F. Wilhelm C. (2021). Effect of stroma on the behavior of temoporfin-loaded lipid nanovesicles inside the stroma-rich head and neck carcinoma spheroids. J. Nanobiotechnology 19 (1), 3. 10.1186/s12951-020-00743-x 33407564 PMC7789590

[B192] Yan C. Zhang Y. Guo Z. (2021). Recent progress on molecularly near-infrared fluorescent probes for chemotherapy and phototherapy. Coord. Chem. Rev. 427, 213556. 10.1016/j.ccr.2020.213556

[B193] Yan J. Gao T. Lu Z. Yin J. Zhang Y. Pei R. (2021). Aptamer-targeted photodynamic platforms for tumor therapy. ACS Appl. Mater. interfaces 13, 27749–27773. 10.1021/acsami.1c06818 34110790

[B194] Yan S. Zeng X. Tang Y. a. Liu B.-F. Wang Y. Liu X. (2019). Activating antitumor immunity and antimetastatic effect through polydopamine-encapsulated core-shell upconversion nanoparticles. Adv. Mater. 31 (46), 1905825. 10.1002/adma.201905825 31566283

[B195] Yang C. Su M. Luo P. Liu Y. Yang F. Li C. (2021). A photosensitive polymeric carrier with a renewable singlet oxygen reservoir regulated by two NIR beams for enhanced antitumor phototherapy. Small 17, e2101180. 34145754 10.1002/smll.202101180

[B196] Yang C. Zhu Y. Li D. Liu Y. Guan C. Man X. (2021). Red phosphorus decorated TiO2 nanorod mediated photodynamic and photothermal therapy for renal cell carcinoma. Small 17, e2101837. 34145768 10.1002/smll.202101837

[B197] Yang D. Dai Y. Liu J. Zhou Y. Chen Y. Li C. (2014). Ultra-small BaGdF5-based upconversion nanoparticles as drug carriers and multimodal imaging probes. Biomaterials 35 (6), 2011–2023. 10.1016/j.biomaterials.2013.11.018 24314558

[B198] Yang L. Shao B. Zhang X. Cheng Q. Lin T. Liu E. (2016). Multifunctional upconversion nanoparticles for targeted dual-modal imaging in rat glioma xenograft. J. Biomaterials Appl. 31 (3), 400–410. 10.1177/0885328216658779 27388895

[B199] Yao J. Huang C. Liu C. Yang M. (2020). Upconversion luminescence nanomaterials: A versatile platform for imaging, sensing, and therapy. Talanta 208, 120157. 10.1016/j.talanta.2019.120157 31816719

[B200] Ye X. Collins J. E. Kang Y. Chen J. Chen D. T. N. Yodh A. G. (2010). Morphologically controlled synthesis of colloidal upconversion nanophosphors and their shape-directed self-assembly. Proc. Natl. Acad. Sci. U. S. A. 107 (52), 22430–22435. 10.1073/pnas.1008958107 21148771 PMC3012508

[B201] Yi Z. Luo Z. Barth N. D. Meng X. Liu H. Bu W. (2019). *In vivo* tumor visualization through MRI off‐on switching of NaGdF _4_ –CaCO _3_ nanoconjugates. Adv. Mater. 31 (37), 1901851. 10.1002/adma.201901851 31364218

[B202] Yin A. Zhang Y. Sun L. Yan C. (2010). Colloidal synthesis and blue based multicolor upconversion emissions of size and composition controlled monodisperse hexagonal NaYF4: Yb,Tm nanocrystals. Nanoscale 2 (6), 953–959. 10.1039/b9nr00397e 20644777

[B203] Yin Z. Zhou D. Xu W. Cui S. Chen X. Wang H. (2016). Plasmon-enhanced upconversion luminescence on vertically aligned gold nanorod monolayer supercrystals. Acs Appl. Mater. Interfaces 8 (18), 11667–11674. 10.1021/acsami.5b12075 27111717

[B204] Yuan J. Cen Y. Kong X.-J. Wu S. Liu C.-L. Yu R.-Q. (2015). MnO2-Nanosheet-Modified upconversion nanosystem for sensitive turn-on fluorescence detection of H2O2 and glucose in blood. Acs Appl. Mater. Interfaces 7 (19), 10548–10555. 10.1021/acsami.5b02188 25919577

[B205] Yuan Y. Gao C. Wang D. Zhou C. Zhu B. He Q. (2019). Janus-micromotor-based on-off luminescence sensor for active TNT detection. Beilstein J. Nanotechnol. 10, 1324–1331. 10.3762/bjnano.10.131 31293869 PMC6604751

[B206] Yuan Z. Fan G. Wu H. Liu C. Zhan Y. Qiu Y. (2021). Photodynamic therapy synergizes with PD-L1 checkpoint blockade for immunotherapy of CRC by multifunctional nanoparticles. Mol. Ther. J. Am. Soc. Gene Ther. 29, 2931–2948. 10.1016/j.ymthe.2021.05.017 PMC8530932 34023507

[B207] Zanger U. M. Schwab M. (2013). Cytochrome P450 enzymes in drug metabolism: Regulation of gene expression, enzyme activities, and impact of genetic variation. Pharmacol. Ther. 138 (1), 103–141. 10.1016/j.pharmthera.2012.12.007 23333322

[B208] Zhang C. Zhao K. Bu W. Ni D. Liu Y. Feng J. (2015). Marriage of scintillator and semiconductor for synchronous radiotherapy and deep photodynamic therapy with diminished oxygen dependence. Angew. Chemie-International Ed. 54 (6), 1790–1794. 10.1002/ange.201408472 25483028

[B209] Zhang G. Li K. He S. Wang L. Guan S. Zhou S. (2021). Electron donor-acceptor effect-induced organic/inorganic nanohybrids with low energy gap for highly efficient photothermal therapy. Acs Appl. Mater. Interfaces 13 (15), 17920–17930. 10.1021/acsami.1c00554 33827214

[B210] Zhang G. Liu Y. Yuan Q. Zong C. Liu J. Lu L. (2011). Dual modal *in vivo* imaging using upconversion luminescence and enhanced computed tomography properties. Nanoscale 3 (10), 4365–4371. 10.1039/c1nr10736d 21904751

[B211] Zhang J. Cheng F. Li J. Zhu J.-J. Lu Y. (2016). Fluorescent nanoprobes for sensing and imaging of metal ions: Recent advances and future perspectives. Nano Today 11 (3), 309–329. 10.1016/j.nantod.2016.05.010 27818705 PMC5089816

[B212] Zhang Y. Schroeder L. K. Lessard M. D. Kidd P. Chung J. Song Y. (2020). Nanoscale subcellular architecture revealed by multicolor three-dimensional salvaged fluorescence imaging. Nat. Methods 17 (2), 225–231. 10.1038/s41592-019-0676-4 31907447 PMC7028321

[B213] Zhang Y. W. Sun X. Si R. You L. P. Yan C. H. (2005). Single-crystalline and monodisperse LaF3 triangular nanoplates from a single-source precursor. J. Am. Chem. Soc. 127 (10), 3260–3261. 10.1021/ja042801y 15755126

[B214] Zhang Z. Shikha S. Liu J. Zhang J. Mei Q. Zhang Y. (2019). Upconversion nanoprobes: Recent advances in sensing applications. Anal. Chem. 91 (1), 548–568. 10.1021/acs.analchem.8b04049 30260218

[B215] Zhang Z. Wang R. Luo R. Zhu J. Huang X. Liu W. (2021). An activatable theranostic nanoprobe for dual-modal imaging-guided photodynamic therapy with self-reporting of sensitizer activation and therapeutic effect. Acs Nano 15 (3), 5366–5383. 10.1021/acsnano.0c10916 33705106

[B216] Zhao S. Tian R. Shao B. Feng Y. Yuan S. Dong L. (2020). One-pot synthesis of Ln(3+)-doped porous BiF3@PAA nanospheres for temperature sensing and pH-responsive drug delivery guided by CT imaging. Nanoscale 12 (2), 695–702. 10.1039/c9nr09401f 31829387

[B217] Zhao Y. He Z. Zhang Q. Wang J. Jia W. Jin L. (2021). 880 nm NIR-triggered organic small molecular-based nanoparticles for photothermal therapy of tumor. Nanomaterials 11 (3), 773. 10.3390/nano11030773 33803677 PMC8003086

[B218] Zhao Y. Yang F. Sun J.-S. Li X.-P. Zhang J.-S. Zhang X.-Z. (2019). Experimental optimal design of Er&lt;sup&gt;3+</sup&gt;/Yb&lt;sup&gt;3+</sup&gt; co-doped Ba&lt;sub&gt;5&lt;/sub&gt;Gd&lt;sub&gt;8&lt;/sub&gt;Zn&lt;sub&gt;4&lt;/sub&gt;O&lt;sub&gt;21&lt;/sub&gt; phosphor and red upconversion luminescence properties. Acta Phys. Sin. 68 (21), 213301. 10.7498/aps.68.20191192

[B219] Zhen X. Cheng P. Pu K. (2019). Cancer phototherapy: Recent advances in cell membrane-camouflaged nanoparticles for cancer phototherapy (small 1/2019). Small 15 (1), 1970002. 10.1002/smll.201970002 30457701

[B220] Zheng F. Xu R. (2020). CircPVT1 contributes to chemotherapy resistance of lung adenocarcinoma through miR-145-5p/ABCC1 axis. Biomed. Pharmacother. 124, 109828. 10.1016/j.biopha.2020.109828 31986409

[B221] Zheng K. Liu X. Liu H. Dong D. Li L. Jiang L. (2021). Novel pH-triggered doxorubicin-releasing nanoparticles self-assembled by functionalized beta-cyclodextrin and amphiphilic phthalocyanine for anticancer therapy. Acs Appl. Mater. Interfaces 13 (9), 10674–10688. 10.1021/acsami.0c19027 33621058

[B222] Zheng S. Chen W. Tan D. Zhou J. Guo Q. Jiang W. (2014). Lanthanide-doped NaGdF4 core-shell nanoparticles for non-contact self-referencing temperature sensors. Nanoscale 6 (11), 5675–5679. 10.1039/c4nr00432a 24769587

[B223] Zhou H. Zeng X. Li A. Zhou W. Tang L. Hu W. (2020). Upconversion NIR-II fluorophores for mitochondria-targeted cancer imaging and photothermal therapy. Nat. Commun. 11 (1), 6183. 10.1038/s41467-020-19945-w 33273452 PMC7713230

[B224] Zhou Y. Chen Y. He H. Liao J. Duong H. T. T. Parviz M. (2019). A homogeneous DNA assay by recovering inhibited emission of rare Earth ions-doped upconversion nanoparticles. J. Rare Earths 37 (1), 11–18. 10.1016/j.jre.2018.05.008

[B225] Zhu S. Yu Q. Huo C. Li Y. He L. Ran B. (2021). Ferroptosis: A novel mechanism of artemisinin and its derivatives in cancer therapy. Curr. Med. Chem. 28 (2), 329–345. 10.2174/1875533xmtazlnzkj1 31965935

[B226] Zou W. Visser C. Maduro J. A. Pshenichnikov M. S. Hummelen J. C. (2012). Broadband dye-sensitized upconversion of near-infrared light. Nat. Photonics 6 (8), 560–564. 10.1038/nphoton.2012.158

[B227] Zou X. Xu M. Yuan W. Wang Q. Shi Y. Feng W. (2016). A water-dispersible dye-sensitized upconversion nanocomposite modified with phosphatidylcholine for lymphatic imaging. Chem. Commun. 52 (91), 13389–13392. 10.1039/c6cc07180e 27786316

